# Evolutionary origin and functional diversification of aminotransferases

**DOI:** 10.1016/j.jbc.2022.102122

**Published:** 2022-06-11

**Authors:** Kaan Koper, Sang-Woo Han, Delia Casas Pastor, Yasuo Yoshikuni, Hiroshi A. Maeda

**Affiliations:** 1 Department of Botany, University of Wisconsin–Madison, Madison, Wisconsin, USA; 2 The US Department of Energy Joint Genome Institute, Lawrence Berkeley National Laboratory, Berkeley, California, USA; 3 Environmental Genomics and Systems Biology Division, Lawrence Berkeley National Laboratory, Berkeley, California, USA; 4 Unaffiliated, Barcelona, Spain; 5 Global Center for Food, Land, and Water Resources, Research Faculty of Agriculture, Hokkaido University, Hokkaido, Japan

**Keywords:** aminotransferases, transaminases, PLP-dependent enzymes, nitrogen metabolism, amino acids, enzyme evolution, AADAT, aminoadipate AT, AGT, alanine:glyoxylate AT, AT, aminotransferase, BCAT, branched-chain amino acid AT, BNA3p, Biosynthesis of Nicotinic Acid protein 3, DAPA, 7,8-diaminopelargonic acid, GABA, γ-aminobutyric acid, GABT, GABA AT, GGAT, glutamate:glyoxylate aminotransferase, GOE, Great Oxidation Event, GOGAT, glutamine oxoglutarate amidotransferase, GS, glutamine synthetase, GTK, glutamine transaminase of kidney or liver, HisP, histidinol phosphate, KAT, kynurenine aminotransferase, LUCA, last universal common ancestor, PDB, Protein Data Bank, PLP, pyridoxal 5′-phosphate, PM, pyridoxamine, PM-AT, PM:pyruvate aminotransferase, PMP, pyridoxamine 5′-phosphate, PPAAT, prephenate AT, PSAT, phosphoserine AT, PYD4, pyrimidine 4, RNP, ribonucleoprotein

## Abstract

Aminotransferases (ATs) are pyridoxal 5′-phosphate–dependent enzymes that catalyze the transamination reactions between amino acid donor and keto acid acceptor substrates. Modern AT enzymes constitute ∼2% of all classified enzymatic activities, play central roles in nitrogen metabolism, and generate multitude of primary and secondary metabolites. ATs likely diverged into four distinct AT classes before the appearance of the last universal common ancestor and further expanded to a large and diverse enzyme family. Although the AT family underwent an extensive functional specialization, many AT enzymes retained considerable substrate promiscuity and multifunctionality because of their inherent mechanistic, structural, and functional constraints. This review summarizes the evolutionary history, diverse metabolic roles, reaction mechanisms, and structure–function relationships of the AT family enzymes, with a special emphasis on their substrate promiscuity and multifunctionality. Comprehensive characterization of AT substrate specificity is still needed to reveal their true metabolic functions in interconnecting various branches of the nitrogen metabolic network in different organisms.

Aminotransferases (ATs), also known as transaminases (Enzyme Commission [EC] 2.6.1.-), are a large family of pyridoxal 5′-phosphate (PLP)–dependent enzymes that catalyze the transamination reactions between amino acid donor and keto acid acceptor substrates (Fig. 1*A*) (1, 2, 3, 4). AT reactions make up ∼50% of all PLP-dependent reactions, which equate to ∼2% of all classified enzymatic activities (5). ATs are functionally diverse and ubiquitous to all kingdoms of life (6, 7). Since free and protein-bound amino acids constitute the predominant form of organic nitrogen, the amino group represents a major nitrogen source for cellular metabolism (8). Consequently, ATs play crucial roles in distributing reduced nitrogen to different branches of both primary and secondary metabolism. For example, plants, fungi, and bacteria assimilate nitrogen in the form of glutamate by glutamine synthetase (GS)/glutamate synthase (also known as glutamine oxoglutarate amidotransferase [GOGAT]) (9, 10, 11) and further distribute the reduced nitrogen to other keto acid acceptors by a variety of ATs, such as aspartate ATs (1, 8). In mammals, alanine AT is a critical enzyme of the alanine–glucose cycle that transports nitrogen and carbon between muscle and liver (12, 13, 14). ATs can also participate in secondary metabolism, such as some isoforms of plant branched-chain amino acid ATs (BCATs) that deaminate methionine to synthesize 4-methylthio-2-oxobutanoate (4MTOB), a precursor of defense compounds, aliphatic glucosinolates (15, 16, 17). In halophilic bacteria, 2,4-diaminobutanoate AT catalyzes the intermediary step of biosynthesis of osmoregulant ectoine from aspartate (18). ATs can also act as conduits between different metabolic branches, such as *Arabidopsis* aromatic AT that transfers amino group from methionine to tryptophan, the precursors of phytohormones, ethylene and auxin, respectively (19). Therefore, AT enzymes participate in various metabolic pathways of amino acids, secondary metabolites, vitamins and cofactors, as well as gluconeogenesis, detoxification, assimilation, and transport of carbon and nitrogen (6, 20, 21, 22).

**Figure 1:**
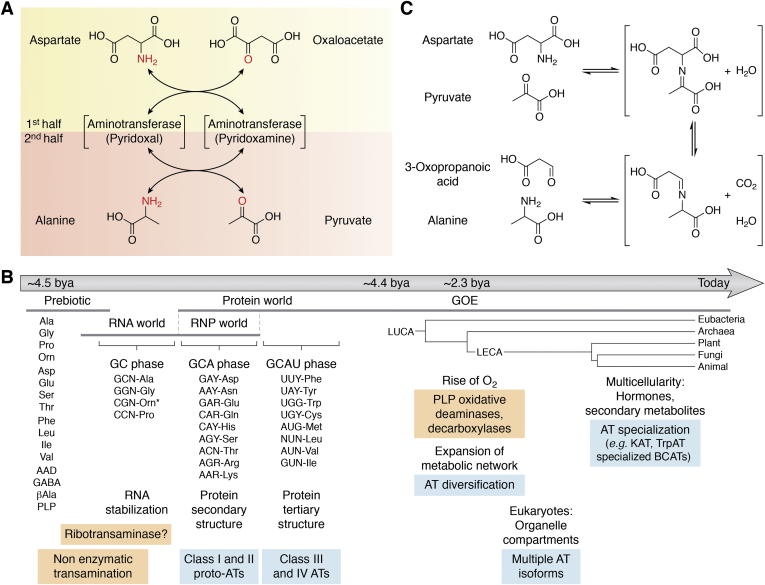
**Enzymatic and nonenzymatic transamination reactions, and the evolutionary history of the amino acid metabolism, proteinogenesis, and transamination.***A*, two half reactions of PLP-dependent transamination. In the first half reaction, an amino group from aspartate is transferred onto PLP, which generates oxaloacetate and PMP. In the second half, the amino group on PMP is transferred to pyruvate, forming alanine and regenerating PLP. *B*, evolution of transamination reactions since the origin of life. Transamination reactions were likely nonenzymatic initially and later catalyzed by hypothetical ribotransaminases during the RNA world, where the two-letter GC coded for a few amino acids. Additional amino acids were recruited after the genetic code expanded to three letters in the GCA phase during the RNA protein (RNP) world, when class I and II proto-ATs might have appeared and streamlined the amino acid metabolism. The subsequent GCAU phase and expansion of proteinogenic amino acids recruited class IV and class III ATs. About 4 billion years ago, LUCA inherited a diverse set of ATs and passed them down to its descendants. ATs underwent additional diversification during and after the Great Oxidation Event (GOE) and the appearance of eukaryotes and multicellularity. *C*, a nonenzymatic transamination reaction between aspartate and pyruvate, where aspartate is converted into an aldehydic acid (3-oxopropanoic acid), rather than keto acid (oxaloacetic acid). AT, aminotransferase; PLP, pyridoxal 5′-phosphate; PMP, pyridoxamine 5′-phosphate; RNP, ribonucleoprotein.

Origins of ATs can be traced back at least to the last universal common ancestor (LUCA), which already had all four distinct AT classes (Fig. 1*B*) (7, 23, 24, 25, 26). Notably, unlike other primary metabolic enzymes, most AT enzymes show substrate ambiguity, which might have originated as an evolutionary constraint because of the mechanistic and structural properties of AT enzymes. Today, the unique versatility of ATs having broad substrate specificity potentially plays important roles in metabolic plasticity and environmental adaption (27). However, the substrate ambiguity can complicate our full understanding of AT enzyme functionality, and there are still many uncharacterized ATs even in model organisms. In addition, multiple, yet unrealized, physiological substrates may still be present for previously characterized enzymes. Despite the critical roles ATs play in the metabolism of all organisms, the most recent reviews on AT enzymes date back to 1990s (2, 3). This review article provides critical updates on this essential enzyme family and highlights new perspectives on AT enzyme evolution and promiscuity and their potential roles in the metabolic network properties.

## Rise of PLP-dependent transamination reaction

All living organisms degrade, interconvert, and synthesize compounds through elaborate chains of chemical reactions to fulfill biological needs, which all together constitutes metabolism (28, 29). The earliest forms of life likely had simpler metabolism consisting of much fewer reactions than extant organisms. The early metabolism expanded over time because of the selective pressure to better utilize new or existing external nutrients, biosynthesize depleted compounds, and generate energy efficiently (28, 29, 30, 31, 32, 33, 34, 35, 36). Biocatalysts were most likely recruited to these metabolic pathways later and accelerated and provided specificity to the chemical reactions (30, 33).

### Nonproteinaceous transamination

Amino acids were likely abundant in the primordial soup and could be abiotically replenished by the conditions present on the early earth (29, 30, 37, 38, 39, 40, 41). Therefore, the earliest forms of life likely used nonenzymatic reactions to metabolize amino acids (Fig. 1*B*) (30, 34, 42, 43, 44, 45, 46, 47, 48, 49, 50, 51). Several nonenzymatic transamination reactions have also been described (43, 52, 53). For instance, Bishop *et al.* (43) showed nonenzymatic decarboxylative transamination between amino and keto acids that generates analogous amino and aldehydic acids (Fig. 1*C*). In addition, PLP is capable of catalyzing nonenzymatic transamination reactions though 10^7^ to 10^9^ times slower than those carried out by PLP-dependent enzymes, where PLP is covalently linked to the active-site lysine *via* a Schiff base (1, 2, 3, 7, 53, 54). Spontaneous formation of pyridoxine-like compounds from components present in the primordial soup is thermodynamically feasible and has been experimentally demonstrated (35, 36). Therefore, it is possible that transamination reactions were initially catalyzed by free PLP, and more complex PLP-dependent apoenzymes might have evolved later, increasing the specificity and catalytic rate (7). As RNA is thought to be an earlier form of biocatalysts (32, 55, 56), the presence of a since-lost ribozyme-mediated PLP-dependent transaminase can be speculated in the RNA world (Fig. 1*B*) (57, 58, 59). However, no natural or artificial “ribotransaminase” is known to date.

### Transition to proteinaceous transamination

**Figure 2:**
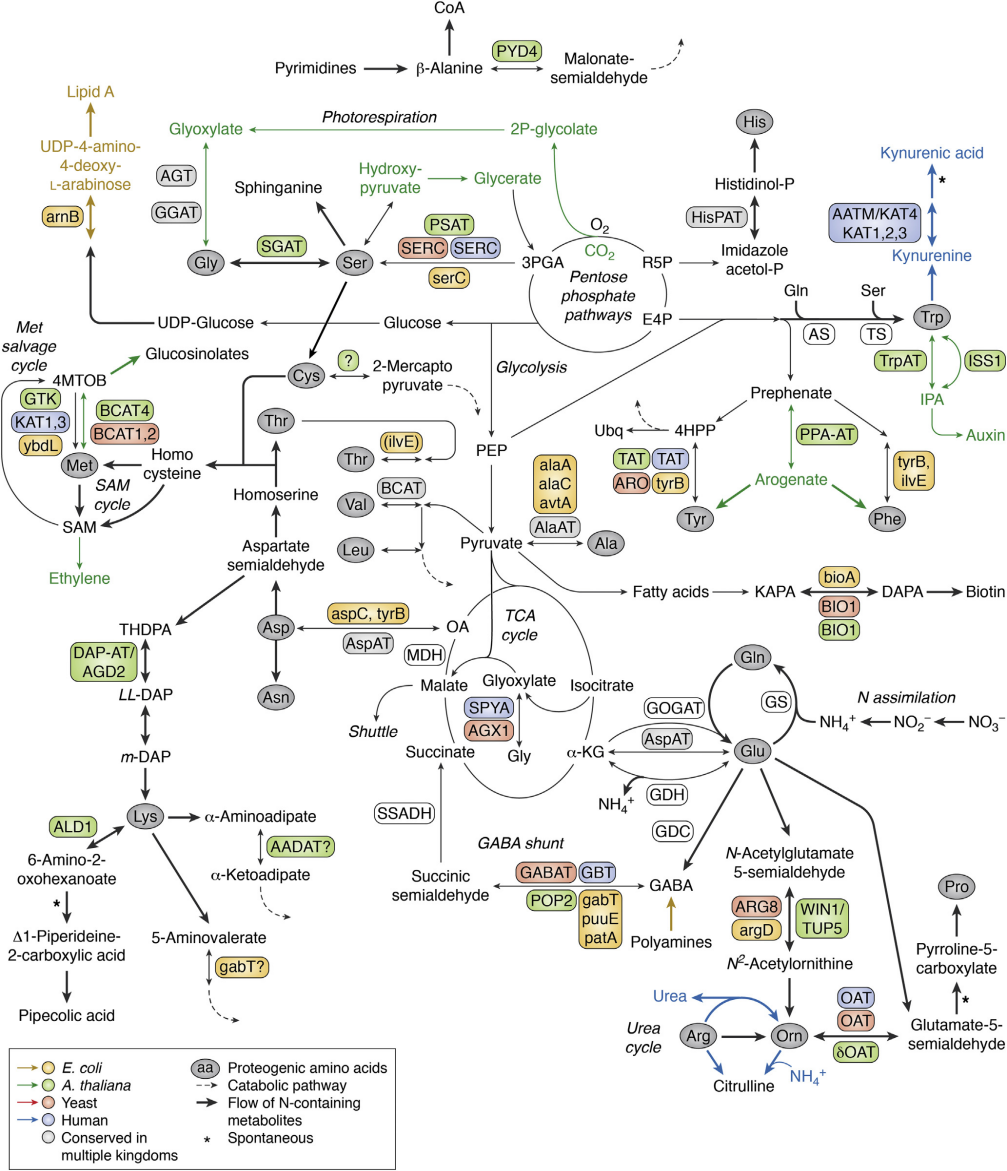
**Metabolic roles of AT reactions in human, yeast, *Arabidopsis*, and *E. coli*.** A metabolic map depicting AT enzyme functions in representative species. Proteinogenic amino acids are shown in *gray ellipses*. *Thick solid and dashed arrows* dictate the flow of nitrogen containing metabolites and catabolic pathways, respectively. ATs and pathways from *E. coli*, *Arabidopsis*, yeast, and human are shown with *yellow*, *green*, *red*, and *blue*, respectively. The AT enzyme abbreviations are listed in Table 1. AS, anthranilate synthase; AT, aminotransferase; DAPA, 7,8-diaminopelargonic acid; E4P, erythrose 4-phosphate; GDC, glutamate decarboxylase; GDH, glutamate dehydrogenase; GOGAT, glutamine oxoglutarate amidotransferase; GS, glutamine synthetase; 4HPP, 4-hydroxyphenylpyruvate; KAPA, 7-keto-8-aminopelargonic acid; α-KG, alpha-ketoglutarate; l,l-DAP, l,l-2,6-diaminopimelic acid; *m*-DAP, meso-2,6-diaminopimelic acid; MDH, malate dehydrogenase; 4MTOB, 4-methylthio-2-oxobutanoic acid; OA, oxaloacetate; PEP, phosphoenolpyruvate; 3PGA, 3-phosphoglyceric acid; R5P, ribose 5-phosphate; SSADH, succinic semialdehyde dehydrogenase; THDPA, tetrahydrodipicolinate; TS, tryptophan synthase; Ubq, ubiquinone.

The transition from an RNA to protein world likely occurred in a stepwise manner through an intermediary ribonucleoprotein (RNP) world (Fig. 1*B*), which is supported by the properties of the genetic code, ribosomes, and some basal proteins (58, 60, 61, 62, 63). The appearance of a protein-encoding system—the genetic code—was likely accompanied by the expansion of the amino acid biosynthesis, in which ATs likely played key roles (58). Hartman and Smith (60, 61) proposed the elegant three-step evolutionary scheme (GC → GCA → GCA/U) for the expansion of genetic code and proteinogenesis (Fig. 1*B*); the very first ribosome-translated proteins were coded by permutations of two nucleotides, guanine (G) and cytosine (C): GG, GC, CC, and CG encoding glycine, alanine, proline, and likely ornithine with a positively charged side chain, respectively. These earliest amino acids could be easily produced from intermediates of central carbon metabolism (*i.e.*, pyruvate and glyoxylate, Fig. 2) (58, 60) and most likely formed random cationic peptides (58, 60) that could interact with and stabilize RNAs. The earlier two-letter coding system is still embedded inside the three-letter codons of glycine (GGN), alanine (GCN), and proline (CCN), where N can be any nucleobase (Fig. 1*B*). Notably, however, CGN today encodes for arginine whose biosynthesis is too complicated to be an early proteinogenic amino acid (Fig. 2). Instead, CGN might have originally coded for an easier-to-produce positively charged amino acid, such as ornithine, a precursor to arginine (58, 60). Ornithine having a shorter side chain than arginine (or lysine) facilitates RNA–peptide interactions and discourages the formation of α-helixes (60, 64). In later evolutionary stages, however, arginine became advantageous as a proteinogenic amino acid over ornithine for the formation of α-helices in protein-based functional folds (60).

Next step in RNP evolution was the recruitment of polar amino acids—aspartate, asparagine, glutamate, glutamine, threonine, serine, histidine, arginine, and lysine—that allowed the peptide portion of the RNP to attain structure (*i.e.*, α-helixes) and participate in catalysis (58). The addition of these new amino acids became possible with the incorporation of adenosine (A), expanding to three-nucleotide codons (*i.e.*, GC**A**) (58, 63) (Fig. 1*B*). All GCA phase amino acids could be produced within a few steps of central metabolism (Fig. 2), except for histidine derived from nucleotides—the precursors for RNA (58)—and lysine, which most likely appeared in late GCA phase (58). Notably, the highly conserved residues of ATs (see “Conserved residues and structural features of AT classes” section) are composed almost entirely of GC and GCA phase amino acids, suggesting that proto-ATs might have appeared during the GCA phase. The early proto-ATs lacking lysine might have employed a PLP–ornithine Schiff base. Alternatively, free vitamin B_6_ might have acted as a cosubstrate, like seen in the extant PLP-independent pyridoxamine (PM) ATs (see “Nitrogen transfer catalyzed by PLP-independent enzymes” section) (65, 66, 67); then PLP became a prosthetic group only after the addition of lysine to the genetic code. Interestingly, promiscuous class I and II ATs could hypothetically catalyze all transamination reactions for AT-dependent GCA phase amino acids (see “Evolution of four classes of AT enzymes” section) and could be the first ATs to emerge (Fig. 2).

Hydrophobic amino acids—leucine, isoleucine, valine, cysteine, methionine, phenylalanine, tyrosine, and tryptophan—were perhaps not proteinogenic in the early GCA phase, since their large nonpolar side chains on a polar amide backbone create intramolecular repulsion that distorts overall structure. However, the establishment of the secondary structure (*i.e.*, α-helixes) by the end of the GCA phase stabilized backbone conformation (58), which set the stage for the introduction of hydrophobic amino acids encoded by GCA**U** that includes uracil (U) (Fig. 1*B*). Since these GCA**U** phase amino acids are synthesized through complex biosynthetic pathways (Fig. 2) (62, 68), the emergence of additional class III and IV proto-ATs during the GCAU phase likely played critical roles in their biosynthesis and recycling pathways, together with the further divergence of the existing class I and II proto-ATs (Fig. 1*B*). Thus, the late emergence of hydrophobic amino acid ATs, such as aromatic ATs and BCATs, may reflect their wide distributions across different AT classes (see “Functional diversification of AT enzymes” section). Hydrophobic motifs were a structural breakthrough allowing proteins to form tertiary folds and enter into the membrane that is inaccessible to RNA (58, 60, 69). Thus, the rise of the GCAU amino acids likely marked the end of the RNA’s dominance and allowed the cellular metabolism to predominantly utilize proteins, except for few indispensable relics of the RNA and RNP worlds, such as the ribosome (70) and spliceosome (71). Around this time, ornithine was likely removed from proteins, as ornithine’s benefits over arginine and lysine for interacting with RNA (64) did not justify an inapt fit for complex protein structures in a “protein world.”

ATs most likely emerged and underwent initial diversification between the GCA phase of the RNP world (Fig. 1*B*) and the time of the LUCA, from which all extant organisms have evolved (25). Recent genome reconstruction studies predicted that all AT classes and the majority of PLP-dependent enzyme types were already found among 355 protein families (57) and 3018 protein-coding genes (25) that were predicted to be present in LUCA. Since the inference of the gene sequences that predate LUCA is inherently imprecise, it is difficult to phylogenetically reconstruct and analyze the initial stages of PLP-dependent enzyme evolution. Nevertheless, a deep analysis of codon structure of ancient PLP-dependent enzymes, similar to studies conducted on aminoacyl-tRNA synthetases and ribosomes (60, 72, 73), could link proteinogenesis and the appearance of proto-ATs.

### Diversification of PLP-dependent enzymes with the rise of oxygen

Although LUCA already had diverse PLP-dependent enzymes and ATs, these and other enzymes further expanded during and after the Great Oxidation Event (GOE), when atmospheric O_2_ levels rapidly increased at ∼2.3 billion years ago (Fig. 1*B*) (74, 75, 76, 77). As the carbanionic intermediate of the PLP-dependent reactions is reactive with oxygen, GOE likely led to the appearance of novel PLP-dependent enzymes that catalyze oxygenic reactions, such as oxidative deamination or decarboxylation (78) (Fig. 1*B*). In bacteria (*i.e.*, *Streptomyces*), PLP-dependent arginine oxidases utilize oxygen in the biosynthesis of heterocycles (78). PLP-dependent amino acid decarboxylases are also reactive with oxygen (78, 79, 80, 81); for example, amino acid aldehyde synthases catalyze oxidative deamination followed by decarboxylation (78), converting phenylalanine to volatile phenylacetaldehyde in petunia flowers (82) and 3,4-dihydroxyphenylalanine to 3,4-dihydroxyphenylacetaldehyde in insects for flexible cuticle formation (83). The increased availability of ATPs through aerobic respiration also made the synthesis of ATP-demanding amino acids (84)—tryptophan, phenylalanine, tyrosine, and arginine—more affordable. Notably, most of the oxygen utilizing PLP-dependent enzymes (*e.g.*, amino acid aldehyde synthase) act on many of those same amino acids (78, 85). Thus, GOE contributed to a tremendous expansion of many metabolic pathways and networks (85), which include biosynthesis of certain amino acids and alkaloids, as well as nitrogen metabolism that incorporated novel PLP-dependent enzymes.

**Figure 3:**
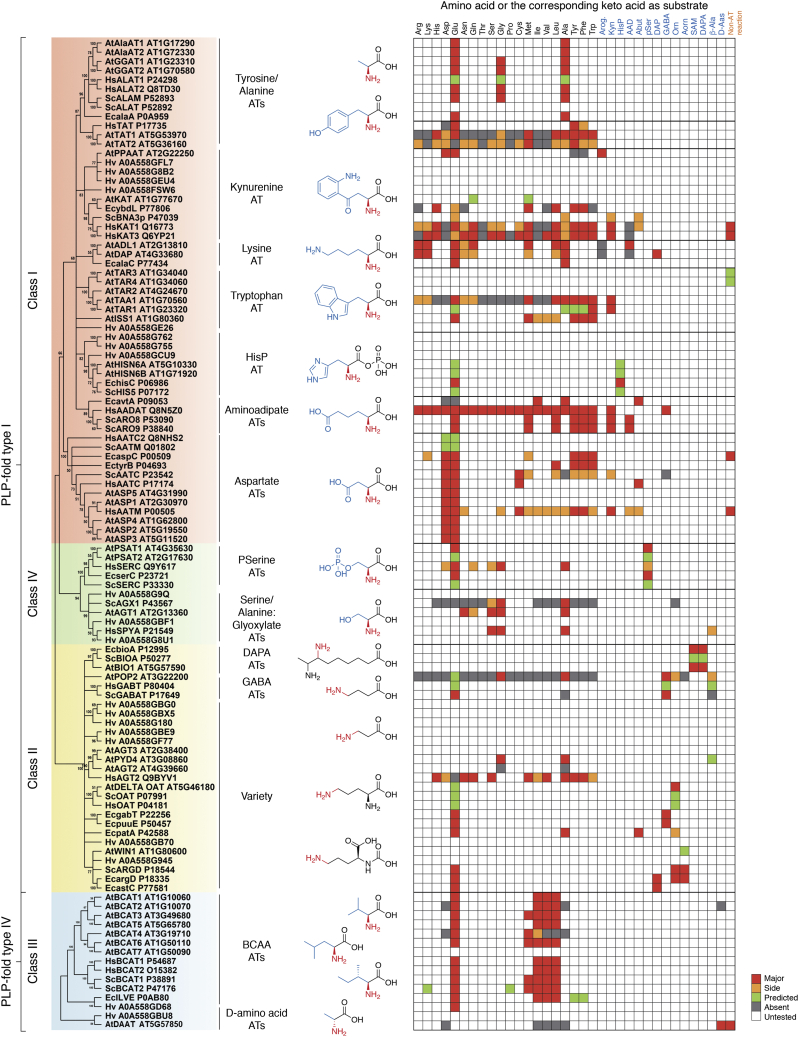
**Phylogeny and reported activities of ATs from representative organisms.** Phylogenetic relationship of ATs from human (Hs), yeast (Sc), *Arabidopsis* (At), *Escherichia coli* (Ec), and *Halobacterium volcanii* (Hv). Multiple sequence alignment was performed using MAFFT-DASH (138), and the analysis was performed under default setting in MEGA 11 (343) neighbor-joining method (344, 345) with partial deletion site coverage set to 50% and 1000 bootstraps (346). Analysis was done separately for class III ATs from class I, II, and IV ATs because of their distinct evolutionary origins. ATs formed at least 12 distinct clades whose major substrates are shown. Activities detected in the literature for each enzyme are shown on the *right*. *Red*, *orange*, *green*, *gray*, and *white* denote major, side, predicted, absent, and untested activities, respectively. AT, aminotransferase.

The expansion of PLP-dependent enzymes after the GOE could also be an indirect consequence of the appearance of complex eukaryotic life derived from the last eukaryotic common ancestor (Fig. 1*B*). For instance, a general expansion of ATs occurred for all eukaryotes, where the appearance of organelles required multiple isoforms in different subcellular compartments (Fig. 1*B*). Later expansion of multicellularity, such as in animals and the land plants, also required ATs with tissue or developmental stage–specific functions (Fig. 1*B*). For instance, in the human brain, kynurenine is converted into kynurenic acid for tryptophan degradation, primarily by kynurenine aminotransferase 2 (KAT2) and to a lesser by KAT1, KAT3, and KAT4 (Fig. 2) (86, 87, 88, 89, 90). While KAT1 and KAT3 are phylogenetically related with each other, KAT2 is closely related to aminoadipate AT and KAT4 to aspartate AT, which gained KAT activity (Fig. 3). *Arabidopsis* plants also have at least three different tryptophan AT isoforms having specific roles: TAA1, TAR1, and TAR2 generate indole-3-pyruvate, the precursor of auxin, in certain developmental stages or tissue types (Fig. 2) (91, 92, 93, 94).

### Nitrogen transfer catalyzed by PLP-independent enzymes

It is important to note that nitrogen transfer reactions can be also catalyzed by PLP-independent enzymes. Pyridoxamine:pyruvate aminotransferase (PM-AT; EC 2.6.1.30) and pyridoxamine 5′-phosphate (PMP):α-ketoglutarate aminotransferase (PMP-AT; EC 2.6.1.54), so far found in bacteria (*i.e.*, *Mesorhizobium loti* (65) and *Clostridium kainantoi* (95)) and plant (*i.e.*, *Nicotiana tabacum* (66)), catalyze reversible transamination reactions between vitamin B_6_ and keto acids in the absence of PLP. PM and PMP, two natural forms of vitamin B_6_, act as amino donors, and their deaminated products are released from the active site, which is contrary to PLP-dependent transamination where PLP functions as a prosthetic group (65, 66, 67, 96). PM-ATs are, however, related to PLP-dependent enzymes based on their similar structural and mechanistic features. For example, the crystal structure of PM-AT from *M. loti* shows typical domain characteristic of fold type I PLP-dependent enzyme and class IV ATs (97), with the presence of a Schiff base linkage of K197-pyridoxal as well as a hydrogen bond network between the enzyme D171 residue and N1 of the pyridine ring as observed in the internal aldimine intermediate of PLP-dependent enzymes. Although the biological roles of PM-AT and PMP-AT are currently unclear, both enzymes are considered to participate in the metabolism of vitamin B_6_ (65, 66).

Glutamine amidotransferases are PLP-independent enzymes that are known to be involved in AT-like nitrogen transfer. However, unlike ATs that have a single active site with the PLP cofactor that directly transfers an amino group from amino and keto substrates, glutamine amidotransferases first hydrolyze the amido group of glutamine at the glutaminase domain and then channel the released ammonia to a separate synthase or transferase domain (98, 99). Glutamate synthase, also known as GOGAT, is an NAD(P)H or ferredoxin-dependent oxidoreductase (EC 1.4.1.14, 1.4.1.13, and 1.4.7.1, respectively) that converts α-ketoglutarate to glutamate using glutamine as amido donor (100, 101). Together with ATP-dependent GS, the GS–GOGAT cycle is responsible for nitrogen assimilation in plants, bacteria, and fungi (Fig. 2) (9, 11, 100). Other glutamine amidotransferases include ATP-dependent asparagine synthetases (EC 6.3.5.4) (102, 103), anthranilate synthase (EC 4.1.3.27) (104), PLP synthase (EC 4.3.3.6) (105), and imidazole glycerol phosphate synthase (EC 4.3.2.10) (106). Interestingly, glutamine-fructose-6-phosphate amidotransferases (EC 2.6.1.16), which are involved with amino-sugar metabolism (107), are competitively inhibited by PLP through its reversible binding to an active-site lysine (pyridoxylation) (108). Amidotransferases are particularly abundant in nucleotide metabolism, such as the purine metabolic enzyme glutamine phosphoribosylpyrophosphate amidotransferase (also known as amidophosphoribosyltransferases or ATase; EC 2.4.2.14) that catalyze the conversion of 5-phosphoribosyl-1-pyrophosphate into 5-phosphoribosyl-1-amine (109).

Certain amino acid dehydrogenases release or incorporate free ammonium ion from or to the Cα of amino acids through NAD(P)^+^/NAD(P)H-dependent oxidative deamination or reductive amination, respectively (110, 111). For instance, glutamate dehydrogenases (EC 1.4.1.2) play critical roles at the interface of carbon and nitrogen metabolism, such as by releasing ammonium ion from glutamate at the entry of the urea cycle (112) or by assimilating ammonium ion to generate glutamate from α-ketoglutarate (111) (Fig. 2). These certain PLP-independent dehydrogenases can transfer free nitrogen between metabolites but cannot catalyze transamination of the α-amino group of amino acids. The apparent indispensable role of PLP in transamination of α-amino group, at least in extant AT enzymes, could come from the hypothesis of “principle of many users” (113, 114, 115), where it becomes very difficult to lose or replace a particular component (*i.e.*, a cofactor) that participates in multiple critical metabolic processes. The replacement of such a component would nullify various critical metabolic pathways, which would significantly decrease fitness (115). Hypothetically, alternative routes could evolve synchronously, but multiple examples suggest that the complete replacement is rare even if the alternative route is more efficient or better suited (*i.e.*, RuBisCO *versus* phosphoenolpyruvate carboxylase) (116). Thus, the PLP cofactor used by modern ATs today likely represents a molecular relic of an ancient metabolic state (59, 113).

## Evolution of four classes of AT enzymes

Modern ATs can be phylogenetically classified into four distinct AT *classes*, three of which (class I, II, and IV) were diversified from an ancestral fold type I PLP-dependent enzyme (Box 1 describing different fold types of PLP-dependent enzymes), whereas AT class III evolved independently from a fold type IV PLP enzyme (Fig. 3) (2, 7, 117).

Box. 1 **Seven distinct fold types of PLP-dependent enzymes**

PLP-dependent enzymes were initially classified into five distinct types based on their overall structure folds that independently adopted PLP through the course of evolution (23). However, as some could not be placed in any of these five types (23), PLP-dependent enzymes are now categorized to seven fold types I to VII (24). AT enzymes are found in the fold type I and IV of PLP-dependent enzymes, which are briefly summarized here.

Fold type I (23) (or α family (7)) is the largest family of PLP-dependent enzymes, mostly ATs (except d-amino acid and BCATs) and some non-AT enzymes involved in amino acid metabolism: amino acid decarboxylases (*i.e.*, prokaryotic ornithinedecarboxylase), aminomutases (*i.e.*, glutamate-1-semialdehyde aminomutase), lyases (*i.e.*, cystathionine-β-lyase), synthases (*i.e.*, DAPA synthase), and hydroxymethyltransferases (*i.e.*, serine hydroxymethyltransferase) (130, 341, 342). Aspartate AT is one of the well-characterized enzymes of this family (Fig. 5) (7, 23). Type I enzymes commonly have their Schiff base lysine closer to the C terminus compared with the glycine-rich region, and a hydrophobic β-strand is present downstream of the lysine (23). An invariant aspartate residue is present 20 to 50 amino acids downstream of the lysine and interacts with the nitrogen of pyridoxal ring (23). In addition, two other residues that interact with PLP are also conserved; an aromatic residue (corresponding to W130 in aspartate AT) and another residue (corresponding to A213 in aspartate AT) that is one of alanine, serine, threonine, valine, isoleucine, proline, or methionine (23). Overall, the structure of type I enzymes consists of a large N-terminal domain of seven-stranded β-sheet and a small C-terminal domain of three or four stranded β-sheet with helices on one side (133, 134). Fold type I enzymes typically form homodimers or homotetramers, which is required for activity as their active site is located at the subunit–subunit interface (133, 134, 135, 136).

Fold type IV (23) (or d-alanine AT family (7)) is another small family containing d-alanine ATs, BCAT, and 4-amino-4-deoxychorismate lyase. d-alanine AT is the prototype enzyme of this family (7). Type IV enzymes consist of two domains of dissimilar sizes. The smaller N-terminal domain contains a six-stranded antiparallel β-sheet that is flanked by two α helices on one side. The larger C-terminal domain is made of four-stranded pseudo-β-barrel with a few surrounding helices and contains the active site lysine (133). Importantly, PLP binds to the active site in opposite direction compared with type I, explaining the unique substrate stereospecificity of type IV enzymes. A glutamic acid molecule interacts with the ring nitrogen of PLP (133). These enzymes are usually active as homodimers, but BCAT further oligomerizes into a hexamer (130, 133).

### Phylogeny of ATs

Structure-guided phylogenetic analysis of 109 AT sequences from representative organisms from different kingdoms—*Homo sapiens* (animal), *Saccharomyces cerevisiae* (fungi), *Arabidopsis thaliana* (plant), *Escherichia coli* (bacterium), and *Halobacterium volcanii* (archaeon)—further supports the evolutionary relationship of four AT classes (Fig. 3) (2, 3, 6, 7, 21). The presence of all four class AT members in all domains of life—Eubacteria, Archaebacteria, and Eukarya—is a testament to the heritage of ATs that can be traced back to LUCA (25, 26) (Fig. 1*B*). Within each class, certain ATs form distinct clades and utilize similar substrates, though there are many exceptions as discussed later (Fig. 3).

Class I is the largest and the most functionally diverse class of ATs and utilizes substrates, such as aspartate, aromatic amino acids, histidinol phosphate, kynurenine, and diaminopimelate (Fig. 3). Class I ATs form a robust monophyletic clade, which also includes certain non-AT enzymes, such as 1-amino-cyclopropane-1 carboxylate synthases (ACC) and some carbon–sulfur lyases (not shown in Fig. 3), involved in the synthesis of ethylene precursor ACC (118) and cysteine metabolism (119, 120), respectively. Class II is a small class of ATs that utilize γ-aminobutyric acid (GABA), ornithine, acetylornithine, 7,8-diaminopelargonic acid (DAPA), and ω-amino acid (Fig. 3). Unlike other classes of ATs that transaminate α-amino/keto groups, class II ATs can act on non-α amino acids (Fig. 3). These ATs are in the same phylogenetic clade with non-AT enzyme, glutamate-1-semialdehyde 2,1-aminomutase, that is involved in the biosynthesis of tetrapyrroles (121). Class IV is another relatively small AT class that utilizes serine and phosphoserine. Non-ATs, cysteine desulfurases, and molybdenum cofactor sulfurase are also found in class IV clade. Class III has d-amino acid and BCATs and is the most structurally distinct AT class that belongs to the independently evolved fold type IV PLP-dependent enzymes (Box 1). This class also contains the non-AT enzyme 4-amino-4-deoxychorismate lyase that is involved with folate biosynthesis. Interestingly, *Arabidopsis* d-amino acid AT also exhibits 4-amino-4-deoxychorismate lyase activity (122, 123).

### Mechanisms of AT-catalyzed reactions

Transamination reactions catalyzed by ATs proceed through two half-reactions employing “ping–pong bi–bi” kinetic mechanism (4, 124, 125). In the first half of the reaction, the PLP at the active site of the enzyme reacts with the amino acid substrate to form PMP, and the corresponding keto acid product is released. The second half of the reaction is essentially the reverse of the first half, where a keto acid substrate reacts with PMP to regenerate PLP and forms the corresponding amino acid (Fig. 1*A*) (4). During the transamination reaction, the cofactor PLP acts as an electron sink, storing electrons from cleaved bonds of the substrate and later dispersing them for the formation of new bonds (4, 6), whereas the protein portion of the ATs (apoenzyme) limits the unintended side reactions and facilitates the main reaction (4, 6, 7, 53).

Most AT-catalyzed reactions are reversible, but sometimes, there is a preferred reaction direction determined by the kinetic properties of the AT enzyme, or through the rapid consumption of one of the end products by a downstream enzyme or process (1, 6, 21, 126, 127). For example, amination of glyoxylate to glycine by glutamate:glyoxylate ATs is physiologically irreversible because of the exceedingly low affinity of glutamate:glyoxylate ATs toward glycine for the reverse reaction (126). Also, deamination of kynurenine by KATs is irreversible since the keto acid product, 4-(2-aminophenyl)-2,4-dioxobutanoate, is unstable and rapidly cyclized to kynurenic acid (Fig. 2).

#### Reaction mechanisms of class I, II, and IV ATs

**Figure 4:**
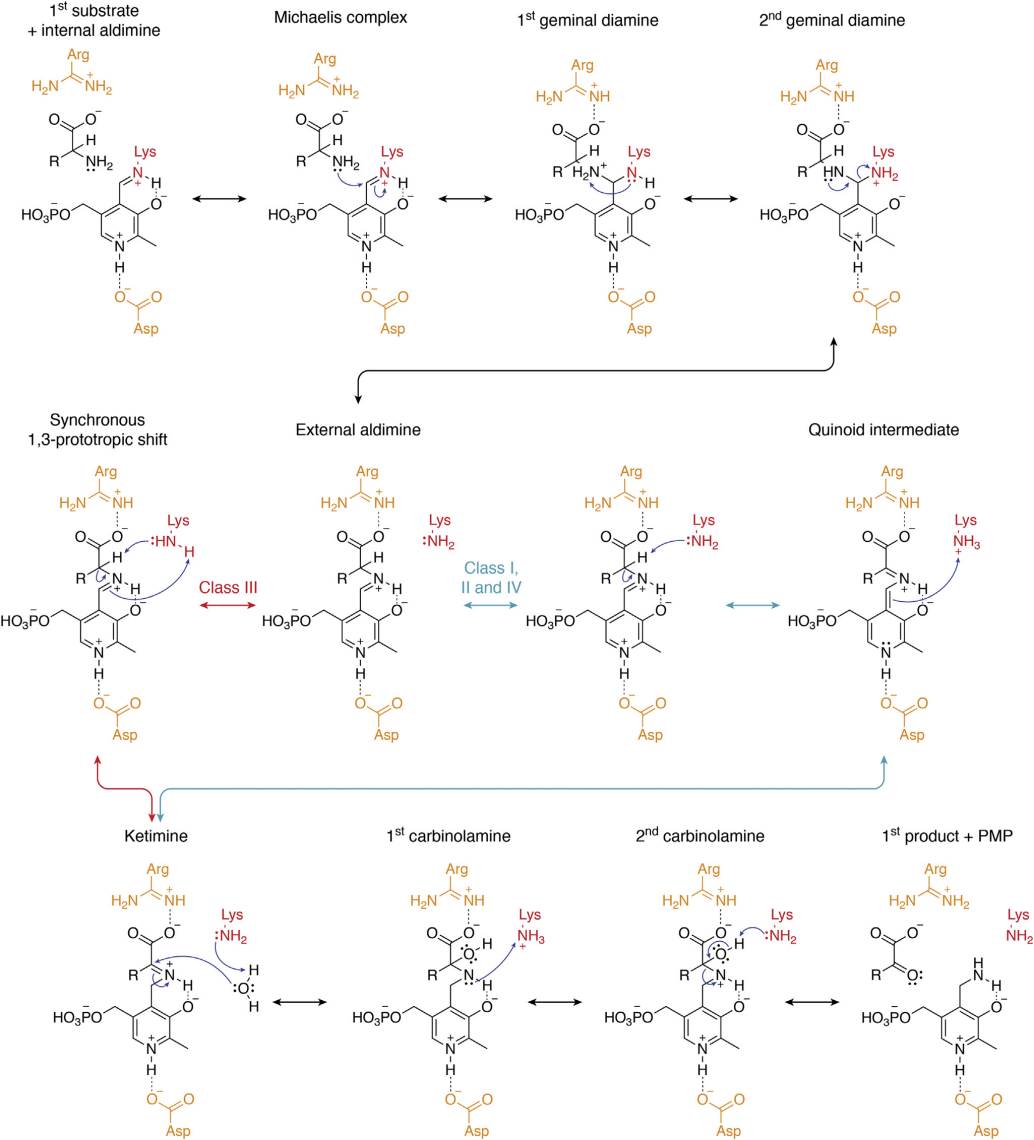
**Reaction mechanisms of AT-catalyzed transamination reaction.** The first half-reaction mechanisms for class I, II, IV, and class III ATs are shown starting from the first amino acid substrate and PLP–enzyme complex (internal aldimine). After the formation of the Michaelis complex, the reaction proceeds through the formation of first and second geminal diamines and external aldimine. In class I, II, and IV ATs, the external aldimine first forms quinoid intermediate, which is subsequently converted to ketamine (*blue arrows*). In contrast, in class III ATs, external aldimine is directly converted to the ketimine through 1,3-prototropic shift mechanism (*red arrows*). Ketimine is subsequently converted to first and second carbinolamine intermediates and collapses to form PMP plus the first keto acid product. In the second half-reaction, PMP reacts with second keto acid substrate in the reverse direction to form second amino acid product and PLP. AT, aminotransferase; PLP, pyridoxal 5′-phosphate; PMP, pyridoxamine 5′-phosphate.

Reaction mechanism of class I, II, and IV ATs is well known; thanks to the detailed characterization of aspartate ATs (4) (Fig. 4). Like other PLP-dependent enzymes, the formation of an internal aldimine in which PLP forms a Schiff-base linkage with an active-site lysine residue is prerequisite for AT activity (128). In the first half-reaction, an unprotonated amino acid substrate binds to a protonated internal aldimine, or a protonated amino acid substrate binds to an unprotonated internal aldimine (4, 124), where the extra proton is mutually transferable. The reactive Michaelis complex requires a protonated internal aldimine and an unprotonated amino group (4, 129), leading to the nucleophilic addition of the substrate amino group to the C4′ of PLP and the formation of the first geminal diamine intermediate. The proton transfer between the two geminal nitrogen further results in the second geminal diamine, which subsequently collapses to form the external aldimine and displaces the active-site lysine as a free base (Fig. 4). Until the external aldimine formation, the reactions are rapid and are often represented as a single step (4).

Next, the external aldimine is deprotonated at the Cα–H bond by the free base lysine and forms the carbanionic intermediate that has three major resonance contributors. The most catalytically potent resonance form is known as the quinonoid intermediate, which has the electron pair from the Cα–H bond delocalized onto the pyridine ring nitrogen. As the reaction can either revert to the original external aldimine or form the ketimine intermediate (*blue arrows* in Fig. 4), a highly conserved aspartic or glutamic acid residue interacts with the ring nitrogen of PLP to keep it protonated and encourages forward progress. After the formation of the ketimine intermediate, a water molecule is added to the Cα by the active-site lysine, which generates the first carbinolamine intermediate. The proton transfer from the lysine to the C4′ nitrogen forms the second carbinolamine intermediate. Finally, the hydroxyl group of the second carbinolamine intermediate is deprotonated by the active-site lysine, and the intermediate collapses into PMP and a free keto acid product (Fig. 4). The second half reaction progresses through the reversal of the first half reaction, where a keto acid substrate reacts with the PMP to form the corresponding amino acid product (4).

#### Reaction mechanisms of class III ATs

Although class III ATs evolved independently from classes I, II, and IV, their reaction mechanisms are remarkably similar because of convergent evolution. This suggests that PLP-dependent transamination likely has certain evolutionary constraints, such as the formation of external aldimine (Fig. 4) (7, 130). Class III transamination follows the same enzymatic steps as class I, II, and IV ATs until the formation of the external aldimine, but how the reaction proceeds forward differs (*red arrows*, Fig. 4). Unlike in classes I, II, and IV ATs, analysis of the *Mycobacterium tuberculosis* IlvE (structural gene E of isoleucine, valine–leucine operon, also known as transaminase B) reaction did not detect the formation of the reactive quinoid intermediate even under short reaction times (∼3 ms) (131, 132). Instead, the reactions go through a synchronous cleavage of the substrate Cα–H bond and the protonation of PLP C4′ *via* 1,3-prototropic shift mechanism to form ketimine intermediate (131). The steps after the ketimine intermediate are identical between classes I, II, IV, and class III ATs (131).

### Conserved residues and structural features of AT classes

**Figure 5:**
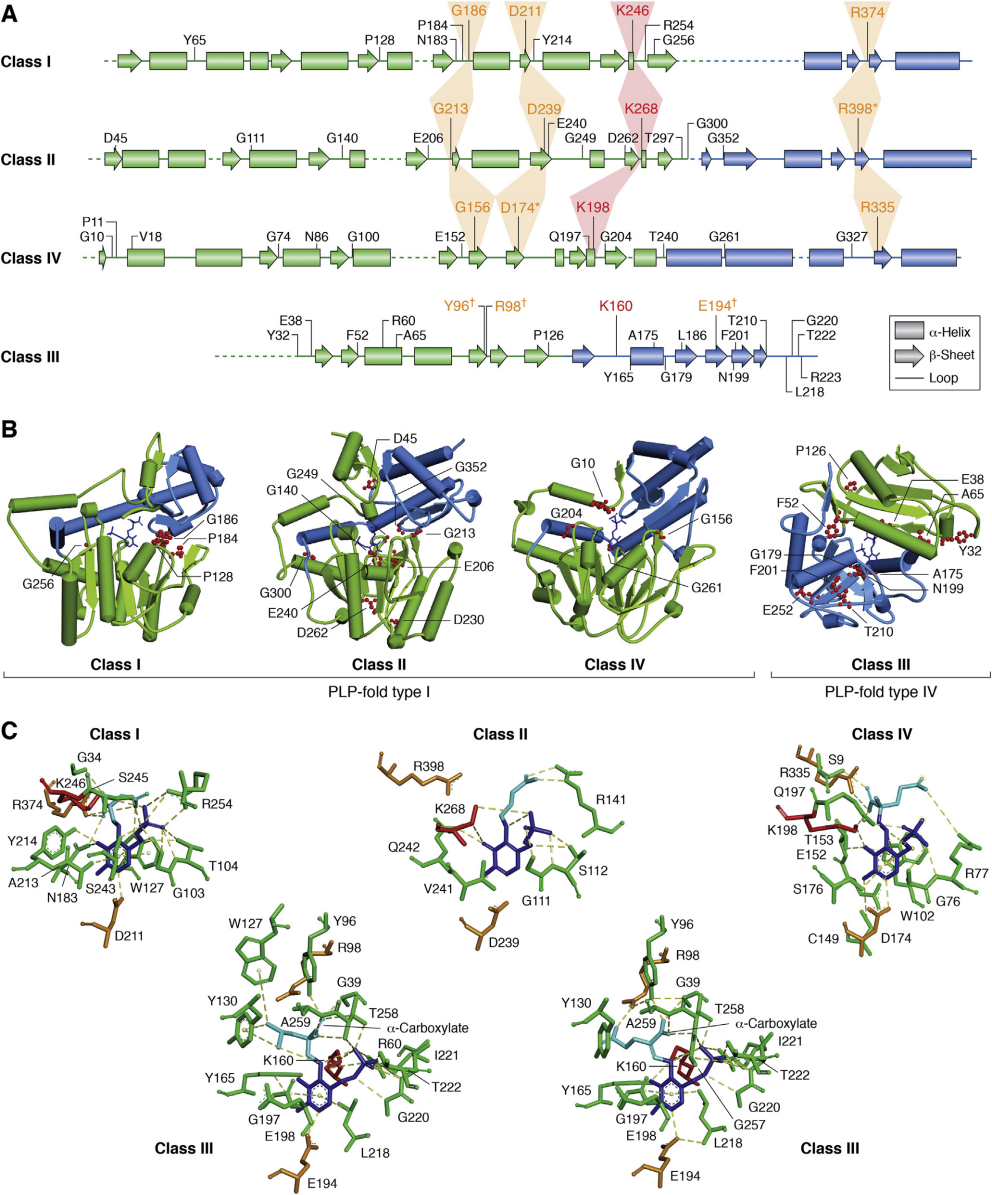
**Structure and conserved residues of four classes of ATs.***A*, simplified secondary structures of AT polypeptides showing the overall topology of each class AT, which include α-helixes (*boxes*), β-sheets (*arrows*), and loops (*straight lines*). *Black residues* are conserved for >90% of aligned sequences within each class, except for the ones marked with a *star*, which are conserved for ∼70%. The Schiff-base lysine is shown in *red* and traced by *redtraces*. Functionally conserved glycine, glutamate/aspartate, and arginine are shown in *orange* and traced by *orangetraces*. Functional conservation of residues marked with Ϯ is inferred from the crystal structure. N- and C-terminal domains are shown in *green* and *blue*, respectively. *B*, overall structures of *Escherichia coli* class I aspartate AT (AAT; Protein Data Bank [PDB] ID: 1ARG (139)), class II GABA AT (GABT; PDB ID: 1SFF (347)), class IV phosphoserine AT (PSAT, PDB ID: 1BJO (141)), and class III BCAT (IlvE; 1i1l (142)). Non–active-site residues that are conserved within each AT class are labeled in *red*. *C*, conserved active-site residues (*green*) of the substrate (*cyan*)–PLP (*dark blue*) complex of four AT classes. The Schiff-base lysine is shown in *red*. IlvE is shown in complexes with both glutamate (*right*) and leucine (*left*). Note that in class I and IV, conserved arginine (R374 in AAT and R335 in PSAT) interacts with α-carboxylate of the substrate. The weakly conserved R in class II (R398 in GABT) does not interact with α-carboxylate of the substrate; instead another conserved arginine (R141 in GABT) fulfills this duty. In class III, a conserved tyrosine (Y96 in IlvE) interacts with α-carboxylate group of both glutamate and leucine, whereas the conserved arginine (R98 in IlvE) only interacts with the acidic side-chain carboxylate of glutamate. AT, aminotransferase; GABA, γ-aminobutyric acid; GABT, GABA AT; PLP, pyridoxal 5′-phosphate.

Class I, II, and IV ATs, which belong to PLP fold type I, share similar topology and three-dimensional structures, whereas class III ATs belong to PLP fold type IV and have distinct structures (Fig. 5). Class I, II, and IV ATs have a large N-terminal domain of seven-stranded β-sheet (*green region* in Fig. 5, *A* and *B*) and a small C-terminal domain of three or four stranded β-sheet with α-helixes on one side (*blue region* in Fig. 5, *A* and *B*) (133, 134). The active site is located at the subunit–subunit interface, whereas the active-site lysine is located at the N-terminal domain (Fig. 5*A*). Furthermore, the active enzymes form dimers or tetramers (133, 134, 135, 136). On the other hand, class III ATs have a smaller N-terminal domain of six-stranded antiparallel β-sheet that is flanked by two α-helices on one side and a larger C-terminal domain of four-stranded pseudo-β-barrel with few surrounding helices (Fig. 5, *A* and *B*). The active-site lysine is located on the C-terminal domain of class III ATs (133) and binds to PLP in the opposite direction compared with class I, II, and IV ATs (Fig. 5*C*), explaining their unique substrate stereospecificity. Class III ATs are usually active as homodimers, but BCATs further oligomerize into hexamer (130, 133) (Box 1). Although the quaternary structure of ATs can affect enzyme-cofactor stability (137), little is known about the dynamics of the quaternary AT structures and if subunits function synergistically or monomers of different ATs can form heteromultimers.

A structure-guided sequence alignment with MAFFT-DASH (138) shows the active-site lysine, which forms a Schiff-base linkage with cofactor PLP (*red* in Fig. 5*A*) is absolutely conserved for all ATs with catalytic activity. Two more active-site residues, aspartate and arginine (*orange* in Fig. 5*A*) are also conserved among all ATs, except for class III that convergently evolved glutamate and arginine (or tyrosine), correspondingly (Fig. 5*A*). The aspartic (or glutamic) acid (*orange* in Fig. 5*C*) interacts with and maintains the protonation of the pyridine ring nitrogen of PLP, which is essential for the forward progress of AT reaction from the external aldimine to ketimine intermediates (see “Mechanisms of AT-catalyzed reactions” section, Fig. 4) (4). The arginine (or tyrosine) residue of class III ATs (*orange* in Fig. 5*C*) coordinates the α-carboxylate of their substrates. Class I, II, and IV ATs, but not class III ATs, also have a structurally conserved glycine (*orange* in Fig. 5*A*), which is found outside the active site and at the interface of N- and C-terminal domains (Fig. 5*B*).

Among class I ATs, seven more residues are highly conserved (∼90%, Fig. 5). N183 and Y214 interact with the pyridine ring, and R254 interacts with the phosphate group of PLP and stabilizes the cofactor (Fig. 5*C*), whereas the other conserved active-site residue, P184, is likely structural (Fig. 5*B*). Y65 at the subunit interface likely also contributes to the stability of PLP, but of the other subunit, through interaction with phosphate groups, whereas P128 and G256 are a part of the solvation shell and core of the enzyme, respectively (Fig. 5*B*). Based on the crystal structure of *E. coli* AAT (or aspC; P00509) (139), additional residues interact with the phosphate group (G103, T104, S243, and S245) and the pyridine ring (W127 and A214) of PLP, though these residues are not highly conserved (∼50–85%) among class I ATs.

Class II ATs have 10 other highly conserved (∼90%) residues (Fig. 5). The active-site residue G111 interacts with the phosphate of PLP (Fig. 5*C*), whereas the two other conserved residues, G140 and E206, are likely structural (Fig. 5*B*). T297 is found at the subunit interface and interacts with the phosphate group from the PLP of the other subunit, whereas G249, D262, and G352 are parts of the solvation shell and D45, E240, and G300 are found at the core of the enzyme (Fig. 5*B*). Based on the crystal structure of *E. coli* GABA AT (gabT, P22256) (140) (Fig. 5), an additional S112 residue interacts with the phosphate group, and V241 and Q242 interact with the pyridine ring of PLP (Fig. 5*C*). These three residues, however, are not highly conserved (∼50–70%) among class I ATs. Interestingly, R398 that is highly conserved among all fold type I ATs has relatively low conservation (∼70%) among class II ATs. For example, DAPA ATs from yeast, *A. thaliana*, and *E. coli* lack R398 that is replaced by a tyrosine residue (140). According to the crystal structure of *E. coli* gabT, the role of R398 in interacting with the substrate could be less strict compared with other fold type I ATs, as the α-carboxylate of a GABA substrate analog, aminooxyacetate, faces away from R398 and, instead, interacts with R141 that is at the opposite end of the active site (Fig. 5*C*). Considering most class II ATs mainly utilize non α-amino/keto acid substrates, such as γ-amino acid (*i.e.*, GABA), β-amino acid (*i.e.*, β-alanine), and diamino acids (*i.e.*, ornithine and DAPA), the substrate α-carboxylate is distantly located from their amino group that attaches to PLP and interacts with a different arginine residue (R141 instead of R398, Fig. 5*C*). The presence of both arginine residues in most class II ATs may allow the use of both α- and non α-amino/keto acid substrates. Mutagenesis and structural studies of different class II AT with both substrate types can further test this hypothesis.

Class IV ATs have 12 additional highly conserved (∼90%) residues (Fig. 5). At the active site, Q197 interacts with the phosphate of PLP (Fig. 5*C*), whereas G10 and P11 are likely structural (Fig. 5*B*). G204 and T240 are at the subunit interface and likely assist dimerization and interact with the phosphate group of the PLP of the other subunit, respectively. G100 and G261 are parts of the solvation shell of the enzyme (Fig. 5*B*). Based on the structure of *E. coli* phosphoserine AT 1 (SerC or Psat; P23721) (141) (Fig. 5), additional active-site residues, G76 and R77, interact with the phosphate, and W102, C149, T153, and S176 interact with the pyridine ring of PLP (Fig. 5*C*), though they are not highly conserved (∼50–80%) among class IV ATs.

Class III ATs have 18 additional conserved residues, which are more than other AT classes. This is likely because of more recent evolution (see “Transition to proteinaceous transamination” section) and less diversification of class III having only two subtypes (*i.e.*, BCATs and d-amino acid ATs). Based on *E. coli* BCAT (IlvE; P0AB80) (142), the E/D194, R98/Y96, and Schiff-base K160 residues, highly conserved among all ATs (Fig. 5), do not align at primary sequences but have identical roles as in class I, II, and IV ATs based on structural analyses (142, 143) (Fig. 5*C*). Notably, however, R98 of class III ATs interacts with the side-chain carboxylate of acidic substrates (144) (*i.e.*, glutamate, Fig. 5*C*) and Y96 instead interacts with the substrate α-carboxylate (142), allowing the use of both acidic and hydrophobic substrates by BCATs (Fig. 5*C*). Y96 is highly conserved among BCATs but not in d-amino acid ATs from *A. thaliana* and *Bacillus* sp. potentially because of the difference in the stereochemistry of d-amino acid substrates. Active-site residues R60, L218, G220, and T222 function for interacting with the phosphate of PLP, Y165 interacts with the ring of PLP, and E38 and N199 are likely structural. L186 is found at the subunit interface, whereas Y32, P126, G179, R223, and E252 are parts of the solvation shell and F52, A65, A175, F201, and T210 are found at the core of the enzyme, respectively. According to the crystal structure of *E. coli* IlvE (P0AB80) (142), additional active-site residues G39, I221, and T258 interact with PLP phosphate group, G197 and E198 interact with PLP ring, whereas Y130, W127, and A259 stabilize the bound substrate. However, these residues are not highly conserved (∼40–80%) among class III ATs.

Among PLP fold type I ATs, analogous roles for several active-site residues could be defined based on spatial configuration. G103 and T104 of class I ATs, G111 and S112 of class II ATs, and G76 of class IV ATs interact with phosphate of PLP (Fig. 5*C*). N183 and N242 of class I and class II ATs, respectively, and A213 and S126 of class I and class IV ATs, respectively, similarly interact with the pyridine ring of PLP (Fig. 5*C*). The aromatic ring W127 and W102 of class I and IV ATs, respectively, facilitate pi stacking with the pyridine ring of PLP (Fig. 5*C*). Overall, conserved residues are concentrated around the AT active site, likely because of the strong selective pressure to maintain the active-site conformation and catalytic activity. However, the conserved residues found inside the core, on the surface, or between monomers point to the importance of maintaining the tertiary and quaternary structures of ATs.

## Functional diversification of AT enzymes

This section will describe various functions of AT enzymes from human (animal), yeast (fungi), *Arabidopsis* (plant), and *E. coli* (bacterium), which represent the majority of published research (145). Although functions of archaeal ATs remain poorly described, we in addition included *H. volcanii* (archaeon) that is a moderate halophile and can grow under conditions similar to *E. coli* and *S. cerevisiae* (146), unlike many other archaea that adapted to extreme environments. We will discuss each AT group, which forms a well-supported phylogenetic clade within each AT class, highlighting their substrate specificity and promiscuity, though their full substrate specificity is still largely uncharacterized (*open* *boxes* in Fig. 3). Representative AT functions and reactions were also mapped onto the metabolic networks (Fig. 2). Different gene/enzyme nomenclatures have been introduced in the literature even for functional orthologs from different organisms, and we kept these original gene/enzyme names here (*e.g.*, Biosynthesis of Nicotinic Acid protein 3 [BNA3p] from yeast and KAT from plants and animals, and ydbL from *E. coli*; all of them belong to the KAT clade, Table 1). A comprehensive dataset on the nomenclature and properties of ATs from human, yeast, *Arabidopsis*, and *E. coli* that are listed in Table 1 is provided at https://nfluxmap.github.io/resources/.

**Table 1:** The list of previously reported AT enzymes from *Arabidopsis*, human, yeast, and *E. coli*

Class	Genes	Associated activity	References
*Arabidopsis thaliana*			
I	ASP1-5	Aspartate AT	(221, 222, 223, 224)
	PPAAT	Prephenate AT	(189, 190)
	TAA1, TAR1-4	Tryptophan AT	(91, 92, 93, 94)
	ISS1	Aromatic amino acid AT	(19, 231)
	TAT1, 2	Tyrosine AT	(21, 164)
	HisN6A, B	Histidinol phosphate (HisP) AT	(195, 196)
	KAT	Methionine AT	(184)
	?	Aminoadipate AT	
	AlaAT1, 2	Alanine AT	(148)
	GGAT1, 2	Glutamate:glyoxylate AT	(155, 156)
	ALD1	Lysine AT	(191)
	AGD2	l,l-Diaminopimelate AT	(192)
II	POP2	γ-Aminobutyric acid (GABA) AT	(260)
	WIN1	Acetylornithine AT	(285)
	δOAT	Ornithine AT	(271)
	BIO1	7,8-Diaminopelargonic acid AT	(251)
	PYD4	β-Alanine AT	(294)
	AGT2, 3	Alanine:glyoxylate AT	(156)
III	BCAT1–7	Branched-chain amino acid/methionine AT	(15, 16, 17, 20, 307)
IV	PSAT1, 2	Phosphoserine AT	(232, 240)
	AGT1/SGAT	Serine:glyoxylate AT	(246, 247)
*Homo sapiens* (human)			
I	AATC, AATM	Aspartate AT	(90, 217)
	ALAT1, 2	Alanine AT	(12, 13, 14)
	TAT	Tyrosine AT	(160, 161, 162, 163)
	KAT1, 3, AATM/KAT4	Kynurenine AT	(88, 167, 168, 169)
	AADAT/KAT2	Aminoadipate AT	(86, 208)
II	GABT	γ-Aminobutyric acid (GABA) AT	(255, 257)
	OAT	Ornithine AT	(274, 275)
	AGT1, 2	Alanine:glyoxylate AT	(287, 288, 289, 290, 291, 292, 293)
III	BCAT1, 2	Branched-chain amino acid AT	(301)
IV	SERC	Phosphoserine AT	(236, 237)
	SPYA	Serine:pyruvate AT	(243)
*Saccharomyces cerevisiae* (yeast)			
I	AATC, AATM	Aspartate AT	(218, 219, 220)
	ALAT, ALAM	Alanine AT	(154)
	HIS5	Histidinol phosphate (HisP) AT	(199)
	ARO8, 9	Aromatic amino acid AT	(182, 203, 204, 205)
II	Uga1	γ-Aminobutyric acid (GABA) AT	(258)
	OAT	Ornithine AT	(274)
	ARG8	Acetylornithine AT	(278, 279)
	BIOA	7,8-Diaminopelargonic acid AT	(252)
III	BCAT1, 2	Branched-chain amino acid/methionine AT	(206)
IV	SERC	Phosphoserine AT	(234)
	AGX	Alanine:glyoxylate AT	(244, 245)
*Escherichia coli*			
I	aspC, tyrB	Aspartate AT	(226, 227)
	tyrB, aspC, ilvE	Tyrosine/phenylalanine AT	(226, 227)
	hisC	Histidinol phosphate (HisP) AT	(200)
	ybdL	Methionine AT	(185, 186)
	alaA, alaC, avtA, ?	Alanine AT	(158)
II	gabT, puuE, patA	γ-Aminobutyric acid (GABA) AT	(264, 265, 266, 267, 268, 269)
	bioA	7,8-Diaminopelargonic acid AT	(253)
	astC, argD	Acetylornithine AT	(281, 282)
III	ilvE	Branched-chain amino acid AT	(308, 309, 310)
IV	serC	Phosphoserine AT	(241, 242)
Sugar AT	arnB	UDP-4-amino-4-deoxyarabinose-ketoglutarate AT	(297)

Since many AT enzymes have multiple EC numbers, the complete list of their activities is provided in Figure 3 and the additional table at https://nfluxmap.github.io/resources/.

### Class I ATs

Class I ATs can be categorized into several phylogenetic clades (Fig. 3) and participate in a wide range of pathways including metabolism of many proteinogenic amino acids (Fig. 2).

#### Alanine/tyrosine AT clade

Alanine/tyrosine ATs make up a functionally diverse clade that mainly contains ATs acting on alanine/glutamate and/or aromatic amino acids, which are generally hydrophobic except for glutamate, a major AT amino donor. Within this clade, the ATs that primarily act on alanine/glycine/glutamate, such as alanine AT and glutamate:glyoxylate AT, are more closely related to each other than to the tyrosie ATs that mainly act on aromatic amino acids (Fig. 3). Interestingly, *H. volcanii* has no ATs in this clade, and these essential activities must be provided by other uncharacterized ATs in this archaeon.

The alanine/glutamate branch of this clade contains human ALAT1 and ALAT2 (also known as SGPT1 and SGPT2; serum glutamate:pyruvate transaminase), yeast ALAT and ALAM (alanine transaminase), *Arabidopsis* AlaAT1, AlaAT2, GGAT1, and GGAT2, and *E. c*oli AlaA. All these enzymes have glutamate:pyruvate or alanine:α-ketoglutarate AT activity and are mostly involved in alanine metabolism to pyruvate, the product of glycolysis, or vice versa (12, 147, 148, 149, 150, 151, 152, 153, 154) (Fig. 2). In animals, alanine ATs are involved in the alanine–glucose cycle, in which alanine produced in the muscle tissue by glutamate:pyruvate AT activity is transported to the liver, where alanine is converted back to pyruvate and glutamate by alanine:α-ketoglutarate AT activity of hepatic ALATs (12, 13, 14). Then, the liver deaminates glutamate for the urea cycle and uses gluconeogenesis to convert pyruvate to glucose, which can be shuttled back to the muscle (14). *Arabidopsis* enzymes also show alanine:glyoxylate (155) and glutamate:glyoxylate activities (156), which are involved in photorespiration and overlap with the activity of class II and class IV alanine:glyoxylate ATs (see later sections). In some C4 plants (*i.e.*, NAD-malic enzyme type carrying out C4 photosynthesis), alanine generated by alanine ATs are transferred from bundle sheath to mesophyll cells as a part of the C4 carbon fixation cycle (157). Notably, extreme redundancy exists in alanine AT activity of *E. coli* as the sextuple mutant of *alaA avtA alaC ilvE tyrB aspC* was still not auxotrophic to alanine (158).

The tyrosine AT branch of this clade contains human TAT and *Arabidopsis* TAT1 and TAT2, which primarily deaminate tyrosine in the initial step of tyrosine degradation (21, 159). Human TAT has narrow substrate specificity and is the only known AT that can discriminate between tyrosine and phenylalanine (160). Although human and other mammalian TATs can also use 3,4-dihydroxyphenylalanine or 3-*O*-methyldopa, their physiological significance is unclear (161, 162, 163). *Arabidopsis* TATs, on the other hand, are highly promiscuous and can utilize other aromatic amino acids (164), which may be involved in rebalancing of aromatic amino acids (21, 165). They can also use methionine efficiently (164), though its metabolic and physiological roles remain to be examined. Notably, non-AT enzymes, such as carbon–sulfur lyases (SUR1 (119) and CORI3 (120)) involved in glucosinolate-specialized metabolism, evolved through recent divergence from TAT enzymes and within the Brassicales (mustard) order (21). To note, *E. coli* and yeast ATs having tyrosine AT activities are found in a different clade of class I ATs (see later).

#### Kynurenine AT (KAT) clade

KAT orthologs are found in all kingdoms (Fig. 3) and characterized by their highly promiscuous nature. Human KAT1 and KAT3 have activity toward glutamine—hence also named as glutamine transaminase of kidney or liver (GTK and GTL), for KAT1 and KAT3, respectively (88, 166)—as well as a wide range of other amino acid substrates, particularly aromatic amino acids and kynurenine (88, 167, 168, 169). In mammals, KATs are involved in the tryptophan catabolic pathway by catalyzing the irreversible transamination of kynurenine to a highly unstable keto acid product, which is spontaneously converted to kynurenic acid (170, 171, 172, 173). Although KAT activity is present in many tissues and cell types (*i.e.*, liver, heart, lungs, leucocytes, astrocytes, and microglia) (166, 170), its role in the brain is particularly important since kynurenic acid is a neuroactive compound that is antagonistic to many excitatory amino acid receptors, such as *N*-methyl-d-aspartate subtype glutamate receptor (174) and α7-nicotinic acetylcholine receptor (170, 171, 172, 173, 175). In the brain, KAT1/GTK acts as methionine AT of the SAM cycle to regenerate methionine from 4MTOB using glutamine as the amino donor (Fig. 2). KAT1/GTK can also prevent buildup of neurotoxic phenylpyruvate by converting it into phenylalanine, which can be ring hydroxylated to tyrosine for degradation (176, 177). Besides their AT activity, KAT1 and KAT3 also has secondary β-lyase activity toward drugs and natural products that contain *S*-conjugates of cysteine and *Se*-conjugates of l-selenocysteine, which may be important for detoxification of halogenated xenobiotics (178, 179, 180).

In yeast, BNA3p shows structural and sequence homology to KATs from other organisms and is demonstrated to have KAT activity *in vitro* (181). However, BNA3p unlikely contributes to KAT activity *in vivo*, as the double knockout of ARO8 and ARO9, aminoadipate AT clade enzymes with KAT activity in yeast (182) (see *later section*), resulted in the complete loss of the apparent KAT activity *in vivo* (181). Further characterization could reveal the function and the main substrate, other than kynurenine, of BNA3p in yeast.

*Arabidopsis* KAT, despite its annotation based on phylogeny (183), has not been demonstrated to have KAT activity. Gene coexpression data indicate that *Arabidopsis* KAT is expressed with the gene encoding 5-methylthioribose kinase of the methionine salvage cycle (184) (Fig. 2). Biochemical characterization of tomato and maize KAT orthologs (initially named after mammalian GTKs) identified 4MTOB and glutamine as the preferred amino acceptor and donor, respectively (184). Therefore, plant KAT orthologs mediate the cryptic methionine AT within the methionine salvage cycle, which is also linked to biosynthesis of a plant hormone, ethylene (Fig. 2) (184). Notably, KATs work together with ω-amidase that converts α-ketoglutaramate, the keto acid of glutamine, into α-ketoglutarate (184), thus directing the reaction toward methionine formation, unlike typical reversible AT reactions. ybdL is KAT homolog in *E. coli*, but KAT activity has not been demonstrated. ybdL mainly functions not only as a methionine AT *in vitro* but also has side activities with histidine, aromatic amino acids, leucine, and glutamine (185, 186). Although its *in vivo* function remains to be examined, ydbL may be also involved in the SAM cycle. Thus, plant KATs and *E. coli* ydbL may be best to be referred to as methionine ATs.

In this clade, *Arabidopsis* in addition has prephenate AT (PPAAT), which converts prephenate into arogenate (187, 188). Arogenate is the immediate precursor for phenylalanine and tyrosine biosynthesis in plants (189, 190), unlike in many microbes, where prephenate is the last common precursor, and hence PPAAT is not required for phenylalanine and tyrosine biosynthesis. Thus, the evolution of PPAAT enzymes rerouted these aromatic amino acid pathways *via* the arogenate intermediate in the plant kingdom (187, 188). Interestingly, *H. volcanii* has four enzymes, three of which are closely related to plant PPAATs, with unknown function (Fig. 3). Some of them may have prephenate AT activity, if *H. volcanii* uses the arogenate pathway for phenylalanine and tyrosine biosynthesis, like in some microbes (190).

#### Lysine AT clade

Lysine AT clade contains *Arabidopsis* ADL1 (AGD2-Like defense response protein 1) and DAP-AT/AGD2 (l,l-2,6-diaminopimelate AT also known as Aberrant Growth and Death 2). Genetic and biochemical evidence support that ADL1 converts lysine to 6-amino-2-oxohexanoate (the keto acid analog of lysine), which spontaneously cyclize into Δ^1^-piperideine-2-carboxylic acid, an intermediate of the plant defense signal pipecolic acid (191) (Fig. 2). Heterozygous *agd2* mutants show mild dwarfism but have elevated levels of defensive compound salicylic acid, whereas homozygous *ald1* mutants showed increased susceptibility to *Pseudomonas syringae* infection (192). Interestingly, homozygous DAP-AT/AGD2 mutation causes embryonic lethality, suggesting an essential role in plant development and likely in lysine biosynthesis (192). ALD1 and DAP-AT could be operating in lysine catabolism and anabolism, respectively (Fig. 2) (192). While *E. coli* alaC phylogenetically associates with the lysine AT clade (Fig. 3), it is involved in alanine biosynthesis (152).

#### Tryptophan AT clade

ATs in the tryptophan AT clade are found almost exclusively in plants and function in deaminating tryptophan into indole-3-pyruvate, which is further converted to a plant hormone auxin, indole acetic acid (91, 193) (Fig. 2). In *Arabidopsis*, tryptophan AT activity has been demonstrated for TAA1 (Tryptophan Aminotransferase of Arabidopsis 1) and TAR1 (Tryptophan Aminotransferase Related 1) and implied for TAR2 based on genetic evidence (92), whereas TAR3 and TAR4, which form a distinct subclade (Fig. 3), remain to be characterized. TAA1 and TAR1 are promiscuous, especially with other aromatic amino acids (91, 92, 93, 94), and are the only known plant ATs having KAT activity. TAR3 and TAR4 have a putative domain of alliinase, the β-lyase involved in the formation of volatile compounds uniquely produced in the genus *Allium*, such as garlic and onions (194). *In vivo* roles of the enzymes in the TAR3 and TAR4 subclade, other than alliinases, are currently unknown.

#### Histidinol-phosphate (HisP) AT clade

Histidinol-phosphate (HisP) ATs act on HisP, a major intermediate of the *de novo* histidine biosynthesis (Fig. 2). ATs in the HisP AT clade are found in all kingdoms except animals, which depend on dietary intake of histidine. Yeast HIS5, *Arabidopsis* HISN6A and HISN6B (195, 196), and *E. coli* hisC are found within this clade. Of the three *H. volcanii* enzymes within the HisP AT clade, the loss of A0A558GCU9 results in histidine auxotrophy (197), like of *Corynebacterium glutamicum* HisC (198) and yeast HIS5 (199). While the activity of yeast, *Arabidopsis*, and *E. coli* HisP ATs with substrates other than HisP is not known (200), HisC of the hyperthermophile *Thermotoga maritima* and *Bacillus subtilis* works as a bifunctional HisP and aromatic AT (201, 202). Therefore, promiscuity might have emerged in some lineages within the HisP AT clade.

#### Aminoadipate AT clade

The aminoadipate AT clade contains promiscuous ATs from yeast, human, and *E. coli*, but not from *Arabidopsis*. Yeast ARO8 and ARO9 show AT activity toward aromatic amino acids, methionine, leucine, kynurenine, and α-aminoadipate (182, 203). ARO8 is involved in the biosynthesis of tyrosine, phenylalanine (182, 203, 204), and lysine (205), and salvage of methionine (206), whereas ARO9 is mainly involved in tryptophan catabolism (203, 204). However, the *aro8* deficiency can be compensated by the presence of ARO9, and only the *aro8/aro9* double mutant show phenylalanine and tyrosine auxotrophy (182, 203). For tyrosine metabolism to 4-hydroxyphenylpyruvate and then eventually to ubiquinone or coenzyme Q biosynthesis (Fig. 2), ARO8 and ARO9, as well as BNA3p, BCATc (or Bat2), and AATC (or Aat2), are redundantly involved based on their quintuple yeast mutant (207). Human AADAT (or KAT2) shows activity toward a wide range of amino and keto acids but uses α-aminoadipate and kynurenine as preferred amino acid substrates (86). In kidney and liver, AADAT/KAT2 deaminate α-aminoadipate into α-ketoadipate as a part of lysine catabolism (86, 208) (Fig. 2). The analogous lysine catabolic pathway exists in plants (209), but the aminoadipate AT activity is likely mediated by an unknown AT(s) from another clade or class. In the brain, AADAT/KAT2 is also important in deaminating α-aminoadipate that is toxic to glial cells and also converts kynurenine to kynurenic acid, together with KAT1 and KAT3 from the KAT clade (see aforementioned section) (86, 210, 211, 212, 213). *E. coli* avtA in this clade is involved in alanine biosynthesis and transaminates pyruvate into alanine using either valine or homoalanine as amino donors (152, 214, 215).

#### Aspartate AT clade

The aspartate AT clade is sister to all other class I AT, and their members are involved in transamination between aspartate and oxaloacetate, which is often coupled to glutamate for further assimilation of nitrogen into aspartate and their derived amino acids (Fig. 2). Aspartate ATs also function as a part of the malate–aspartate shuttle, where oxaloacetate is reduced by NADH and malate dehydrogenase to malate, which can then be transported across organelle compartments as a reducing equivalent (Fig. 2) (216), as seen for human cytosolic AATC and mitochondrial AATM (217). In addition, AATM is able to irreversibly catalyze the synthesis of kynurenic acid from kynurenine in the brain and hence has the alternative name of KAT4 (90). Humans also have one additional aspartate AT homolog, AATC2, that however lacks the conserved active-site arginine and remains to be functionally characterized. Yeast has a similar pair of aspartate ATs, AATC and AATM, which localize to peroxisome and mitochondria, respectively, and are also involved in the malate–aspartate shuttle based on biochemical and genetic evidence (218, 219, 220).

*Arabidopsis* contains five aspartate AT homologs (ASP1–ASP5) with different subcellular localizations: mitochondrial ASP1, cytosolic ASP2 and ASP4, and plastidial ASP3 and ASP5 (221, 222, 223). ASP2 plays the major role in nitrogen assimilation and transport, as the *asp2* loss-of-function mutant shows 80% reduced aspartate transported in the phloem with compromised growth (222, 224). Other *asp* mutants of *Arabidopsis* show little change in amino acid profile or growth suggesting their functional redundancy (222).

*E. coli* has two enzymes in this clade aspC (also known as AAT) and tyrB, which are likely derived from a shared ancestral enzyme through a recent duplication (225). In fact, aspC/AAT has high *K*_*M*_ and low activity toward aromatic amino acids tyrosine and phenylalanine (226, 227), and substantial tyrosine AT activity can be obtained by DNA shuffling and *in vivo* selection of AspC/AAT (225, 228). aspC/AAT oversees aspartate synthesis and coordinate cell cycle in *E. coli* (229). TyrB exhibits AT activity toward tyrosine as well as other aromatic amino acids and (3*S*)-2-oxo-3-phenylbutanoate (230). The triple mutants of tyrB, aspC, and ilvE (a class III enzyme) are auxotrophic to tyrosine and phenylalanine, suggesting that these enzymes from three distinct clades are together responsible for tyrosine AT activity in *E. coli* (226), similar to the situation in yeast (207). Interestingly, *H. volcanii* has no aspartate AT homolog and may have a novel aspartate AT(s) in other AT clades or classes.

#### Other class I ATs

Class I contains several ATs that form single enzyme clades, which appear to have distal evolutionary relationship to other class I ATs. *Arabidopsis* aromatic amino acid AT, ISS1, clearly belongs to class I but does not associate with any specific sister clade and shows activity with a variety of aromatic amino acids (19, 231). The phenotype of the *iss1* mutant supports that, together with TAA1 and TAR enzymes from the tryptophan AT clade, ISS1 plays a role in homeostasis of tryptophan and its derived hormone auxin *in planta* (19, 231). A *H. volcanii* enzyme A0A558GE26 in class I does not associate with any sister clade either (Fig. 3), and its function is currently unknown.

### Class IV ATs

Class IV contains alanine/serine AT and serine/phosphoserine AT clades (Fig. 3) and are mainly involved in the metabolism of serine, glycine, and alanine.

#### Phosphoserine AT clade

The phosphoserine AT clade contains enzymes from human (SERC), yeast (SERC), *Arabidopsis* (PSAT1 and PSAT2), and *E. coli* (serC), but none from *H. volcanii*. These enzymes catalyze the reversible glutamate:3-phosphohydroxypyruvate transamination that forms *O*-phospho-l-serine in the phosphorylated pathway of serine biosynthesis (Fig. 2) (232, 233, 234, 235). Human SERC (also known as PSAT) plays a critical role in the brain, since serine cannot be transported efficiently across the blood–brain barrier (236, 237). Indeed, mutations in *SERC* lead to phosphoserine AT deficiency (PSATD) and Neu-Laxova syndrome 2 (237, 238). *Arabidopsis* has two isoforms, PSAT1 and PSAT2, both of which localize to the plastids and are involved in serine biosynthesis *via* the phosphoserine pathway (Fig. 2) (232). Although photorespiration is the major source of serine in photosynthetic tissues (239), silencing of PSAT1, but not PSAT2, resulted in the strong growth retardation, which can be rescued by serine supplementation, highlighting the importance of nonphotorespiratory synthesis of serine *via* PSAT1 (232). The PSAT1-silenced plants also showed disturbed ammonia assimilation in roots likely because of reduced recycling of α-ketoglutarate to be used in the GS–GOGAT cycle (232, 240). In addition to serine biosynthesis, *E. coli* serC can also catalyze glutamate:3-hydroxy-4-phospho-hydroxy-α-ketobutrate transamination that forms *O*-phospho-4-hydroxy-l-threonine for the synthesis of its own cofactor PLP (241, 242).

#### Alanine/serine AT clade

The alanine/serine:glyoxylate AT clade contains human SPYA (serine:pyruvate AT), yeast AGX1 (alanine:glyoxylate aminotransferase), and *Arabidopsis* AGT1 (alanine:glyoxylate aminotransferase), which are not closely related to functionally similar class I alanine AT and glutamate:glyoxylate ATs or class II β-alanine/l-alanine ATs from *Arabidopsis* and human. Human SPYA is present in peroxisomes and involved in detoxification of glyoxylate to glycine. The loss-of-function mutations of *SPYA* lead to the hereditary kidney stone disease, primary hyperoxaluria type 1, because of increased excretion and accumulation of oxalate from glyoxylate oxidation (243). Yeast AGX1 converts glyoxylate to glycine using alanine as an amino donor and participates in glycine biosynthesis, as a side branch of the glyoxylate shunt (Fig. 2) (244, 245). *Arabidopsis* AGT1 (or serine-glyoxylate AT [SGAT]) can catalyze transamination reactions between various substrates, such as serine:glyoxylate, alanine:glyoxylate, serine:pyruvate, and asparagine:glyoxylate AT activities (246, 247). AGT1/SGAT is localized in the peroxisome and is essential for photorespiration in photosynthetic tissues (246). Although serine is its preferred amino donor, AGT1 can also use asparagine (248) and is involved in metabolism of serine, glycine, and asparagine in roots (247, 249). This clade contains no enzymes of *E. coli*, as most bacteria primarily convert glyoxylate to malate but has three uncharacterized *H. volcanii* enzymes, two of which are closely related to human SPYA (Fig. 3).

### Class II ATs

Human, yeast, *Arabidopsis*, and *E. coli* ATs within class II can be categorized into several clades based on their phylogenetic relationship (Fig. 3). AT reactions catalyzed by class II ATs are mainly involved with the metabolism of nonproteinogenic amino acids (Fig. 2).

#### 7,8-Diaminopelargonic acid (DAPA) AT (BIO1) clade

7,8-Diaminopelargonic acid (DAPA) ATs are involved in biosynthesis of biotin, an essential enzyme cofactor known as vitamin B_7_, and are found in yeast (BIOA), *Arabidopsis* (BIO1), and *E. coli* (bioA) but not in animals that lack biotin biosynthesis. In plants and fungi, BIO1 is a part of a bifunctional enzyme and fused with a dethiobiotin synthetase (also known as BIO3) (250, 251, 252), whereas in bacteria, BIO1 and BIO3 are separate enzymes (253). Yeast, *Arabidopsis*, and *E. coli* BIO1 catalyzes the conversion of 7-keto-8-aminopelargonic acid to DAPA (Fig. 2), using specifically SAM as the amino donor. DAPA AT is the only AT known to use SAM as an amino donor to date (250, 251, 252, 253), but some bacterial DAPA ATs can utilize other unusual amino donors such as lysine, instead of SAM (*i.e.*, *B. subtilis*) (254).

#### γ-Aminobutyric acid (GABA) AT clade

GABA ATs form a small clade that contains yeast GABAT and human GABT and transaminates GABA to succinic semialdehyde (255, 256). However, *Arabidopsis* and *E. coli* GABTs (*i.e.*, AtPOP, EcgabT, EcpuuE, and EcpatA, Fig. 3) are not part of the GABA AT clade (see *later section*). Human GABT, also known as ABAT, is an enzyme of the GABA shunt that deaminates and controls the level of the neurotransmitter GABA (255) (Fig. 2). Human GABT is also able to use β-alanine as a substrate (257), a reaction that does not occur in yeast GABAT (256). Yeast GABAT, also known as Uga1 (Utilization of GABA 1), deaminates GABA as a source of nitrogen (258), which can also enhance oxidative stress tolerance by increasing the NADPH pool through succinic semialdehyde dehydrogenase (GABAT in Fig. 2) (259).

#### Other class II ATs

In addition, class II contains a variety of ATs with different metabolic roles that do not confidently associate with a certain clade. Interestingly, though, they all have activities with nonproteinogenic and nonalpha amino acids (Fig. 3).

*Arabidopsis* and *E. coli* also have GABA ATs but distantly related to yeast GABAT and human GABT (Fig. 3). *Arabidopsis* POP2 is localized in mitochondria (260, 261) and recycles amino acids and nitrogen *via* the GABA shunt pathway together with glutamate decarboxylase and succinic semialdehyde dehydrogenase (succinic semialdehyde dehydrogenase, Fig. 2) (262, 263). POP2 specifically uses GABA as the only amino donor among 21 other amino acids tested, whereas both pyruvate and glyoxylate, but not α-ketoglutarate, can act as amino acceptors (260). In contrast, *E. coli* gabT is required for GABA utilization as its sole nitrogen source (264) and also act as the 5-aminovalerate AT for degradation of lysine (265). puuE also deaminates GABA but is induced in the presence of putrescine, which can be converted to GABA and used as the sole nitrogen source (266). Putrescine can be also degraded by patA through an alternative pathway *via* γ-aminobutyraldehyde, which can be spontaneously cyclized to Δ^1^-pyrroline or enzymatically oxidized to GABA (267, 268). patA also transaminates other alkane–α-ω-diamines, including the cadaverine and spermidine for polyamine degradation (268, 269) (Fig. 2). *E. coli* puuE, patA, and gabT are closely related phylogenetically and likely evolved from a promiscuous ancestral enzyme but have been co-opted to function in different steps of polyamine and GABA metabolic pathways (Fig. 3).

Ornithine AT enzymes catalyze the conversion of ornithine to glutamate-5-semialdehyde (270, 271), which spontaneously cyclizes into pyrroline-5-carboxylate (271), an intermediate of arginine and proline metabolism (272, 273) (Fig. 2). While human and yeast OATs function in the synthesis of pyrroline-5-carboxylate (274, 275), *Arabidopsis* δOAT is involved in the recycling of nitrogen during arginine catabolism *via* ornithine (271). Although the diamino acid ornithine has an α-amino group, ornithine ATs transaminate the side-chain δ-amino group but not the α-amino group (Fig. 3).

Acetylornithine ATs convert *N*-acetylglutamate-5-semialdehyde into *N*^*2*^-acetylornithine (276), as a part of *de novo* arginine biosynthesis (277, 278, 279) (Fig. 2). Yeast ARG8 is essential for arginine biosynthesis (278, 279), and *E. coli*, argD is a bifunctional enzyme having both acetylornithine AT and *N*-succinyl-l,l-diaminopimelate:α-ketoglutarate AT activities involved in arginine and lysine biosynthesis, respectively (280). In contrast, *E. coli* astC, paralogous to ArgD (Fig. 3), utilizes succinylornithine and ornithine as substrates (281, 282) and functions in arginine catabolism *via* the arginine succinyltransferase pathway (283, 284). Phylogeneticall related *Arabidopsis* WIN1 (HopW1 Interacting Protein 1 also known as Tumor Prone 5, TUP5, Fig. 3) can complement the arginine auxotrophy of yeast ARG8 mutant (285), and its *Arabidopsis* mutant exhibits reduced arginine accumulation. Therefore, WIN1/TUP5 is likely responsible for the acetylornithine AT activity in plants, which has been detected in soybean plastid fraction (286), though its biochemical characterization remains to be conducted.

β-Alanine/l-alanine AT subclade is represented by human AGT2 and *Arabidopsis* AGT2, AGT3, and PYD4 (pyrimidine 4). Human AGT2 is a promiscuous mitochondrial enzyme that was first discovered to be an AGT (287) but can use a number of other substrates including *N*^*G*^*,N*^*G*^-asymmetric dimethylarginine (ADMA), a potent inhibitor of renal nitric-oxide synthase essential for the regulation of blood pressure by kidneys (288, 289, 290, 291). Human AGT2 also acts as d-β-aminoisobutyrate:pyruvate AT for thymine catabolism (292, 293). The function of *Arabidopsis* AGT2 and AGT3 is poorly understood, and recombinantly expressed AGT2 lacks alanine:glyoxylate AT activity (156). PYD4 coexpresses strongly with PYD1, PYD2, and PYD3 of the reductive pyrimidine nucleotide degradation pathway and is predicted to catalyze the β-alanine AT step (thus also known as BAT) (294, 295, 296). PYD4 showed alanine:glyoxylate AT activity *in vitro*, although its physiological significance is unclear (294), and can also functionally complement alanine and β-alanine deficient *E. coli* mutants (294). Importantly, both human and *Arabidopsis* harbor another alanine:glyoxylate AT from class IV—human SPYA and *Arabidopsis* AGT1 (Fig. 3, see “Class IV ATs” section)—, which is more active in glyoxylate metabolism than class II alanine:glyxylate ATs.

*H. volcanii* has seven enzymes in class II but, except for one (A0A558G945) related to acetylornithine ATs, none are closely related to enzymes from other organisms (Fig. 3). Thus, ATs in this family might have diverged relatively recently, perhaps, at least after the divergence of archaea and eubacteria.

### Other PLP fold type I ATs

Sugar ATs, like *E. coli* UDP-4-amino-4-deoxy-l-arabinose:oxoglutarate AT (arnB), form a functionally and structurally distinct AT group within PLP fold type I enzymes (Box 1). These sugar ATs are typified by nucleotide interacting loops that are not found in other types of ATs (297, 298, 299). arnB homologs from other bacteria have been characterized as sugar ATs that produce amino-sugars, such as UDP-4-amino-4-deoxy-l-arabinose (297), which is incorporated into lipid A from the outer membrane lipopolysaccharide (300) (Fig. 2). *H. volcanii* also contains a sugar AT, annotated as A0A558GCH4, with unknown function. Because of the distal evolutionary relationship of sugar ATs to other PLP fold type I ATs, they are not included in the phylogeny in Figure 3.

### Class III ATs

Class III ATs mainly contain d-amino acid AT and BCAT enzymes (Fig. 3) and are mainly involved with the metabolism of amino acids with unusual stereochemistry, such as branched-chain and d-amino acids (Fig. 2).

#### Branched-chain amino acid AT (BCAT) clade

BCATs found in this clade are present in all five kingdoms and transaminate valine, leucine, and isoleucine. However, some yeast and *Arabidopsis* BCAT isoforms can also utilize methionine (15), whereas *E. coli* IlvE can utilize phenylalanine (226).

In human, BCATs are catabolic enzymes that degrade the essential branched-chain amino acids and synthesize glutamate, although the reaction is generally reversible. Human BCAT2 is mitochondrial and thus is also known as BCATm (301). However, one splice variant of BCAT2 lacks the signal peptide and is cytoplasmic (302), whereas another variant has a 12-amino acid deletion and is localized in nuclei and mitochondria (303). Human BCAT1 is cytoplasmic (hence BCATc) and is mostly present in the peripheral nervous system of brain, ovaries, and testes (301). In the rodent brain, BCATc and BCATm are both involved in nitrogen transfer between astrocytes and neurons where branched-chain amino acids are used as amino donors for neurotransmitter biosynthesis (304, 305, 306).

Yeast BCAT1 (mitochondrial, also known as Bat1p) is involved in the synthesis of branched-chain amino acids and also of methionine from 4MTOB in the methionine salvage pathway using branched-chain amino acids as amino donors (206) (Fig. 2). Yeast BCAT2 (cytoplasm, also known as Bat2p) mainly functions in the degradation of branched-chain amino acids and methionine salvage pathway. BCAT2 is more promiscuous than BCAT1 and uses branched-chain amino acids as well as lysine and proline as amino donors (206).

*Arabidopsis* has seven BCAT paralogs, though BCAT7 has no detectable transcript and is likely a pseudogene (20, 307). The remaining six BCATs include mitochondrial BCAT1, plastidial BCAT2, BCAT3, BCAT5, and cytosolic BCAT4, BCAT6 (20, 307). All of them, except BCAT4, show strong BCAT activity *in vitro* and are able to rescue the yeast auxotrophic mutant of branched-chain amino acids (307). The mitochondrial and plastidial BCATs are typically involved in the catabolism and biosynthesis of BCAAs, respectively (307). Notably, BCAT4 shows the highest activity toward 4MTOB and 4MTOB, intermediates of the methionine-derived glucosinolate biosynthesis, and only has residual activity with leucine and its keto acid, 4-methyl-2-oxopentanoic acid (16). BCAT3 and BCAT6 also have substantial activity toward 4MTOB, the keto acid of methionine (15, 17). Thus, BCAT3, BCAT4, and BCAT6 are also involved in methionine metabolism (Fig. 2 pathway) as their knockout mutants of *Arabidopsis* have altered levels of methionine and methionine-derived specialized metabolites, such as glucosinolates (15, 16, 17).

*E. coli* ilvE reversibly catalyzes the final steps of isoleucine, valine, and leucine biosynthesis and can also catalyze transamination of tyrosine and phenylalanine though inefficiently (308, 309, 310). *H. volcanii* BC61-TAm is most closely related to *E. coli* ilvE, although the biological function is unclear. Whereas BCAT activity has not been tested, this archaea enzyme can utilize (*R*)-methylbenzylamine as an amino donor with α-ketoglutarate being the acceptor (311). Consistent with the lifestyle of halophilic mesophile *H. volcanii*, BC61-TAm is most active at 50 °C with 1 M NaCl (146, 311).

#### d-Amino acid AT clade

d-Amino acids are the d-enantiomers of proteinogenic amino acids that are found on microbial cell walls and certain bioactive peptides like bacterial antibiotics and the venoms and toxins of various animals (312). d-Amino acids are typically produced from their l-enantiomers by racemases (313). *Arabidopsis* and *H. volcanii*, but not yeast, human, or *E. coli*, have ATs in this d-amino acid AT clade that are the only representative of this clade. However, d-amino acid AT orthologs are present in certain bacteria (*i.e.*, *Bacillus* species (314)) and fungi (*i.e.*, *Aspergillus* species (315)). *Arabidopsis* DAAT is the major AT involved in metabolism of d-amino acids, which stimulate ethylene production through unknown mechanisms (123). *Arabidopsis* DAAT prefers d-methionine among many other d-amino acid substrates that can be used and utilizes both pyruvate and, to a lesser extent, α-ketoglutarate as amino acceptors (forming d-alanine and d-glutamate, respectively) (123, 316). *Arabidopsis* DAAT also functions as 4-amino-4-deoxychorismate lyase for the synthesis of a folate precursor, aminobenzoate (122). *E. coli* facilitates d-amino acid metabolism by racemases that can interconvert l- and d-amino acids (317), whereas yeast and humans use d-amino acid oxidases to deaminate d-amino acids (318).

## Potential modes and mechanisms of AT diversification

An interesting property of many ATs is their apparent substrate promiscuity even from the incomplete biochemical data (Fig. 3). For instance, *Arabidopsis* TAT1 catalyzes transamination between glutamate and tyrosine but can also use phenylalanine, tryptophan, histidine, methionine, and leucine (Fig. 3) (164). Similarly, human KAT2 prefers kynurenine and aminoadipate as substrates but can also use 16 other amino acids (86). Substrate specificity can differ considerably, even among closely related ATs, and often accompanies only subtle differences in active-site conformation that affect a substrate-binding and a catalytic process (319). Consequently, mutations of active-site residues can significantly influence catalytic properties of ATs and drive AT evolution (320, 321, 322, 323).

### Mechanical and structural basis of AT promiscuity

During AT-catalyzed reactions, two substrates, often with different chemical properties or structures, need to bind to the same location on the active site sequentially (as discussed in “Mechanisms of AT-catalyzed reactions” section). In most organisms, nitrogen shuttles are constructed around glutamate (30, 37), aspartate (324), and alanine (325), since their corresponding keto acids—α-ketoglutarate, oxaloacetate, and pyruvate, respectively—are gluconeogenic and abundant intermediates of glycolysis and tricarboxylic acid cycles, which allow efficient coregulation of carbon and nitrogen metabolism. Therefore, many ATs evolved to utilize at least one of these three amino/keto acids as substrates. For example, in plants, assimilated ammonia is transferred mainly as glutamate or aspartate, and the amino group is transferred to other keto acid substrates by ATs (1, 8, 10). Similarly, in animals, excess amino acids are first converted to glutamate by different ATs and subsequently deaminated oxidatively by glutamate dehydrogenase to yield ammonia (326). Consequently, many ATs that act on uncharged hydrophobic (*e.g.*, leucine) and aromatic (*e.g.*, tyrosine) substrates must also be able to bind to a negatively charged hydrophilic substrate (glutamate).

AT active sites utilize two mechanisms to accommodate two or more substrates having side chains of different sizes and chemical properties (327). In one example, aromatic amino acid ATs induce a rearrangement of the hydrogen bond network through conformational changes, allowing the formation of charged pockets for acidic side chains and neutral pockets for aromatic side chains at the same location (327). In the case of BCATs, however, a hydrophobic pocket that is implanted with hydrophilic islands behaves much like a “lock-key” model and is capable of housing both hydrophobic and acidic side chains without undergoing conformational change (142, 319, 327). It is important to note that these two mechanisms are not mutually exclusive, and many other ATs, such as *E. coli* HisP AT and *Thermus thermophilus* glutamine AT and acetylornithine AT, can achieve multisubstrate specificity by a mixture of the two solutions (142, 327, 328, 329).

**Figure 6:**
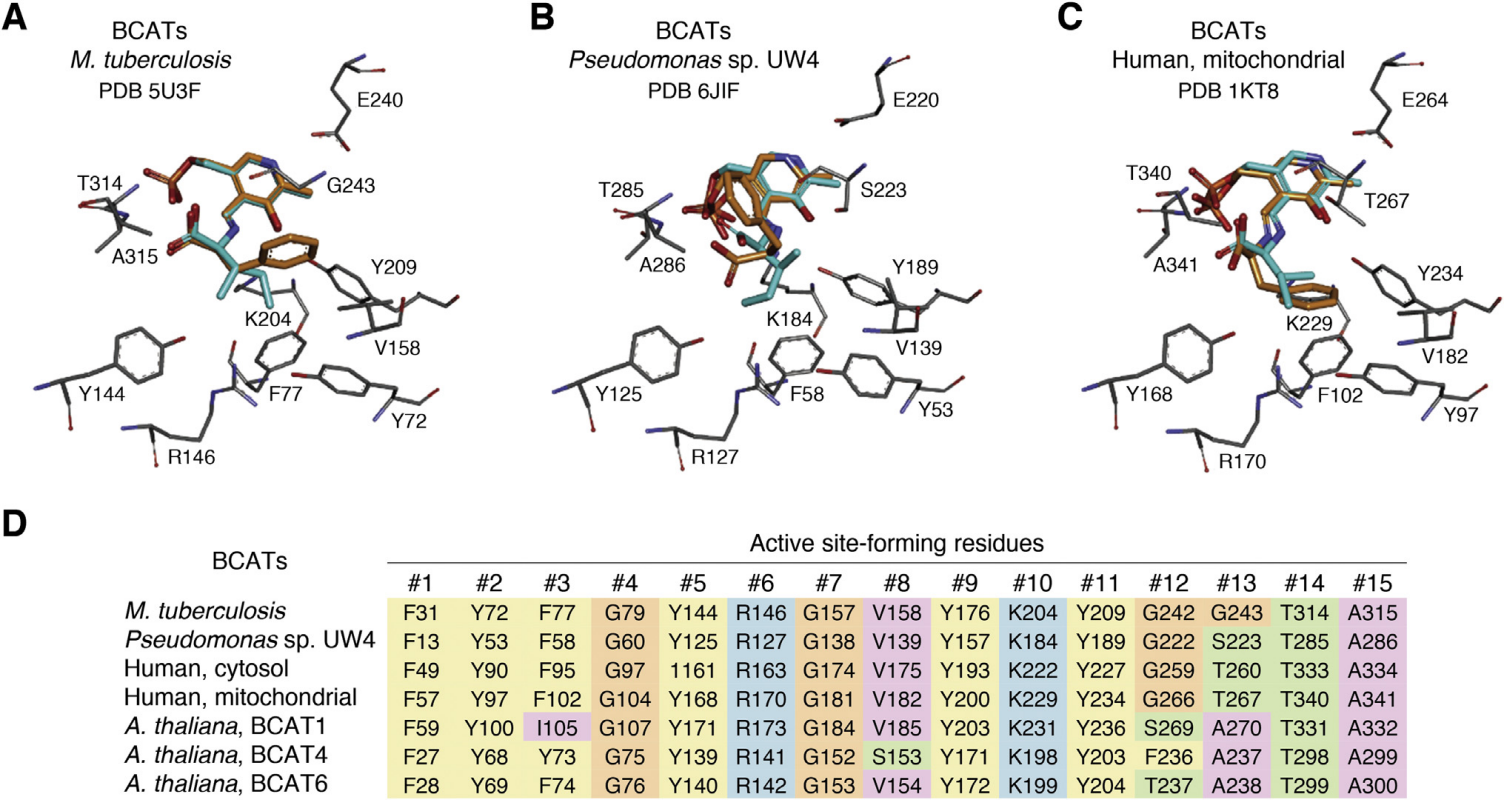
**Sequence and structure features determining the substrate specificity of BCATs.** Molecular modeling to predict binding modes of external aldimine intermediates of isoleucine (*cyan*) and phenylalanine (*orange*) with three homologous BCATs: *A*, *Mycobacterium tuberculosis* (PDB ID: 5U3F). *B*, *Pseudomonas* sp. UW4 (PDB ID: 6JIF). *C*, human mitochondria (PDB ID: 1KT8). *D*, putative amino acid residues of several BCATs consisting of the active sites deduced from sequence alignment. Color shading indicates physicochemical properties of amino acids: aliphatic/hydrophobic (*pink*), aromatic (*yellow*), glycine (*orange*), hydrophilic (*green*), and positive (*blue*). BCAT, branched-chain amino acid AT; PDB, Protein Data Bank.

Computational approaches such as molecular modeling have been employed to comprehend the molecular basis of substrate promiscuity (130). While the structural basis of multisubstrate specificity achieved by conformational rearrangement of the active site remains difficult to simulate without crystallization of individual enzymes at different reaction stages, enzymes that use the “lock–key” mechanism, such as BCATs, can be computationally simulated with reasonable accuracy. In our protein–ligand docking analyses of external aldimine intermediates with isoleucine and phenylalanine, *M. tuberculosis* BCAT (Protein Data Bank [PDB] ID: 5U3F, Fig. 6*A*) showed nearly the same binding orientation regardless of substrates, whereas *Pseudomonas* BCAT (PDB ID: 6JIF, Fig. 6*B*) and human mitochondrial BCAT (PDB ID: 1KT8, Fig. 6*C*) with phenylalanine, but not isoleucine, triggered a tilt on the entire intermediate structure presumably induced by S223 and T267, respectively (#13 position in Fig. 6*D*). This result suggests that the tilt can deteriorate the proton abstraction by 1,3-prototropic shift, an important mechanism to proceed transamination reactions in BCATs (131), by weakening the interaction of pyridine ring N-E264 and the H-bond networks between Cα-H, pyridine ring O, catalytic Lys, and Y234 of the human mitochondrial BCAT (Fig. 6*C*). The sequence analysis of active-site residues in BCATs supports the modeling result and shows the highest divergence at the residue involved (*i.e.*, position #13 that corresponds to T267 of human mitochondrial BCAT, Fig. 6*D*).

Structural and/or mechanistic changes could easily eliminate promiscuity of one half-reaction, such as in the case of aminoacyl-tRNA amidotransferase GatCAB that has separate binding sites for the amido donor glutamine and amido acceptor Glu-tRNA^Gln^, and uses an ammonia channel to transfer and form Gln-tRNA^Gln^ (330). While glutamine and asparagine are structurally and chemically very similar, *k*_cat_/*K*_*M*_ of GatCAB for glutamine is ∼140-fold higher than asparagine (331). However, if an AT gained a similarly high preference for a substrate of one-half reaction, the active site could inadvertently not be able to effectively recognize the substrate of the other half-reaction, which would reduce fitness. Therefore, the evolution of AT substrate specificity is shaped by a tight balance to bind the substrates of both half-reactions. The tradeoff could be the inability to prevent side reactions of substrates that are chemically or structurally similar to, or in-between of, the main substrates. Indeed, multisubstrate specificity is observed for many ATs, especially with related substrates, for example, tyrosine, phenylalanine, and tryptophan used by many aromatic amino acid ATs, and alanine, serine, and glycine utilized by alanine:glyoxylate ATs (15, 21, 246, 247). Thus, both mechanistic and functional constraints likely contributed to the apparent substrate promiscuity of ATs, which in turn provided a unique opportunity for ATs to mediate multiple reactions *in vivo*, unlike often highly specific primary metabolic enzymes.

### At multisubstrate specificity for metabolic plasticity and robustness

The substrate promiscuity of ATs is not necessarily a drawback and can provide advantages in new or changing environments (321, 322). Many AT reactions with secondary cosubstrates could provide metabolic plasticity, when the availability of the main cosubstrate is low, preventing a certain AT-catalyzed reaction to seize up. For instance, TAA1 can continue to deaminate tryptophan into indole-3-pyruvate for auxin production even in the limited availability of its main amino acceptor pyruvate (91), such as under hypoxia with active glycolysis, as TAA1 can also use phenylpyruvate or 4-hydroxyphenylpyruvate (94). Collectively, the substrate promiscuity or side activities of many AT enzymes could form an “underground” (320, 322) amino acid metabolic network, which may have negligible influence on overall metabolism under optimal conditions but can rebalance amino acid levels, such as under various stresses. Therefore, the multisubstrate specificity of AT enzymes may play key roles in metabolic plasticity and robustness in response to environmental changes.

AT substrate promiscuity could lead to multifunctionality if a certain side activity confers enhanced fitness and is positively selected for while also maintaining primary activity. For instance, human KAT2, or AADAT, mainly functions as detoxifying aminoadipate in the liver and kidney (86, 208) while degrading both aminoadipate and kynurenine in the brain (see “Class I ATs” section) (86, 210, 211, 212, 213). Since yeast aminoadipate ATs (ARO8 and ARO9) are also promiscuous with aromatic amino acids including kynurenine (182), the promiscuous kynurenine AT activity was likely present in an ancestral eukaryotic aminoadipate AT and positively selected for to have a role in the brain, while maintaining the primary aminoadipate AT activity. Ultimately, the ability to act on many substrates is advantageous for detoxification enzymes like KAT2 (332, 333)

It is important to note, however, that AT substrate promiscuity can be selected against in some cases. Certain substrates should not be consumed by ATs in other pathways, even when the alternative substrate is structurally or chemically similar. For example, human TAT, unlike other tyrosine ATs (see “Class I ATs” section), is highly specific toward tyrosine but not phenylalanine (160), which only differ by a 4-hydroxy group on the aromatic ring. While humans cannot synthesize any of the aromatic amino acids, tyrosine is deemed conditionally essential because excess phenylalanine is converted to tyrosine by phenylalanine 4-hydroxylase (334, 335). Thus, human TAT, which mainly functions to breakdown excess tyrosine (160), needs to reject phenylalanine not to inadvertently deplete the phenylalanine pool and interfere with phenylalanine 4-hydroxylase to produce tyrosine.

## Conclusion and future perspectives

Transamination reactions have always been an essential part of the cellular metabolic network. Pre-LUCA life may have initially achieved transamination nonenzymatically, or through free PLP, followed by nonproteinogenic biocatalysts (*i.e.*, ribotransaminase), which were then gradually replaced by proteinogenic ATs. ATs evolved independently from two distinct PLP-dependent enzyme fold type I (for class I, II, and IV ATs) and fold type IV (for class III ATs). AT family enzymes were already diversified before LUCA and further expanded during and after the GOE with the appearance of complex eukaryotic life. Reconstructing and characterizing ATs at ancestral nodes, including those of LUCA and last eukaryotic common ancestor, can potentially recapitulate evolutionary pathways of AT enzymes and the nitrogen metabolic networks throughout life history.

Class I, II, IV ATs and class III ATs convergently evolved active sites that can catalyze AT reactions using nearly identical mechanisms, likely because of the universal utilization of PLP in biological transamination reactions. The ping–pong bi–bi mechanisms of AT reactions between two distinct substrates structurally predisposed ATs to maintain substrate promiscuity, which provided a starting point for the evolution of new enzymes and pathways (*i.e.*, biosynthesis of new amino acids and specialized metabolites). Although AT reaction mechanisms have been previously characterized in detail (4), the structural basis of the promiscuity is not yet fully understood. AT enzymes crystallized with a diverse set of substrates could reveal structural features that influence substrate promiscuity, which in turn can facilitate computational prediction and redesign of the function of ATs. In addition, detailed characterization of the subunit–subunit dynamics of AT multimers could further reveal some of the poorly understood properties of ATs, such as subunit synergy and potential heteromultimer formations between different ATs (137, 336, 337).

Many AT enzymes have been characterized, but the functions of some AT enzymes are still unknown even in model organisms (*i.e.*, human, yeast, *Arabidopsis*, *E. coli*). Given that a certain AT reaction can be mediated by ATs from different clades or even different classes depending on organisms, predicted functions of AT enzymes from nonmodel organisms based solely upon sequence alignments should be considered tentative and confirmed experimentally. Considering the rapid evolvability of ATs because of their inherent substrate promiscuity, unlike other primary metabolic enzymes, it would be interesting to examine potential differences in AT substrate specificity between different species even within the same kingdom (*e.g.*, C3 *versus* C4 plants having different nitrogen use efficiency, carnivorous *versus* herbivorous animals having different dietary nitrogen) and assess their impacts on overall nitrogen metabolic network.

Since most studies “looked for” AT enzymes that can catalyze a certain reaction of one’s interest, the potential side activities of AT enzymes have not been tested in many cases. These promiscuous AT reactions may be physiologically insignificant under optimal growth conditions but can provide metabolic plasticity and robustness such as under changing environmental conditions. Testing all substrate combinations—that is, 20 amino acid and 19 keto acid substrates give 380 combinations—is challenging with traditional methods; therefore, the development of high-throughput methods capable of characterizing the full spectrum of AT reactivity is needed. In the meantime, computational approaches, such as molecular modeling (338, 339) and deep/machine learning (340), can accelerate the functional mapping and sequence–structure–function analyses of ATs. Together, comprehensive characterization of AT substrate specificity will reveal the true functionality of AT enzymes and their roles in interconnecting different branches of nitrogen metabolic networks, which are surely much more complex than we currently understand.

## Conflict of interest

The authors declare that they have no conflicts of interest with the contents of this article.
